# Printing Double-Network Tough Hydrogels Using Temperature-Controlled Projection Stereolithography (TOPS)

**DOI:** 10.1021/acsami.3c04661

**Published:** 2023-06-15

**Authors:** Puskal Kunwar, Bianca Louise Andrada, Arun Poudel, Zheng Xiong, Ujjwal Aryal, Zachary J. Geffert, Sajag Poudel, Daniel Fougnier, Ivan Gitsov, Pranav Soman

**Affiliations:** † Biomedical and Chemical Engineering Department, Syracuse University, Syracuse, New York 13210, United States; ‡ BioInspired Institute, Syracuse, New York 13210, United States; § Department of Mechanical and Aerospace Engineering, Syracuse University, Syracuse, New York 13244, United States; ∥ Department of Chemistry, State University of New York ESF, Syracuse, New York 13210, United States; ⊥ The Michael M. Szwarc Polymer Research Institute, Syracuse, New York 13210, United States

**Keywords:** double-network hydrogel, projection stereolithography, additive manufacturing, digital micromirror, mechanically reconfigurable soft devices

## Abstract

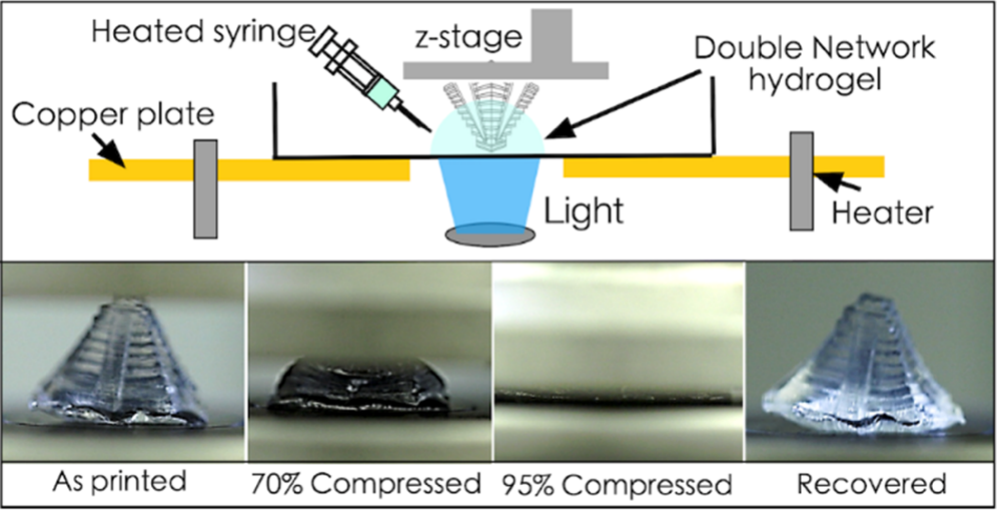

We report a new method to shape double-network (DN) hydrogels into customized 3D structures that exhibit superior mechanical properties in both tension and compression. A one-pot prepolymer formulation containing photo-cross-linkable acrylamide and thermoreversible sol–gel κ-carrageenan with a suitable cross-linker and photoinitiators/absorbers is optimized. A new TOPS system is utilized to photopolymerize the primary acrylamide network into a 3D structure above the sol–gel transition of κ-carrageenan (80 °C), while cooling down generates the secondary physical κ-carrageenan network to realize tough DN hydrogel structures. 3D structures, printed with high lateral (37 μm) and vertical (180 μm) resolutions and superior 3D design freedoms (internal voids), exhibit ultimate stress and strain of 200 kPa and 2400%, respectively, under tension and simultaneously exhibit a high compression stress of 15 MPa with a strain of 95%, both with high recovery rates. The roles of swelling, necking, self-healing, cyclic loading, dehydration, and rehydration on the mechanical properties of printed structures are also investigated. To demonstrate the potential of this technology to make mechanically reconfigurable flexible devices, we print an axicon lens and show that a Bessel beam can be dynamically tuned via user-defined tensile stretching of the device. This technique can be broadly applied to other hydrogels to make novel smart multifunctional devices for a range of applications.

## Introduction

Hydrogel materials have found applications in drug delivery, tissue engineering, biosensing, soft robotics, flexible electronics, and soft photonics. However, traditional single-network hydrogels typically exhibit inferior mechanical properties, which limit their use in the field.^1−5^ To address this challenge, double-network (DN) hydrogels have been developed for applications that require superior toughness, stretchability, and compressive strength.^6−13^ DN hydrogels typically consist of two entangled networks that can be polymerized using two independent stimuli; one network allows energy dissipation during deformation, while the other network provides toughness and/or stretchability.^6,11−16^ Many one-pot synthesis strategies (sol–gel transitions, click chemistries, sequential polymerization) have been used to synthesize DN gels; however, shaping them into customized 3D structures remains a significant challenge.^7−9,17−23^ Conventional molding and casting methods are used to generate simple geometries such as sheets, slabs, and disks,^8,24,25^ while extrusion-based 3D printing has also been used to print customized 3D shapes, although at low resolution and speeds.^7,26−28^ Light-based printing methods can print at high resolutions, and the ability to rapidly photo-cross-linking with DN hydrogels remains a significant materials challenge.^20,29−32^

Bottom-up projection stereolithography (PSLA) has emerged as the favorite light-based 3D printing method due to its capability to make customized parts with microscale resolution and superior design flexibility.^33−36^ A typical setup consists of spatially modulated light patterns projected through a transparent bottom window to cross-link photosensitive liquid resins in *XY* plane before moving the stage up (*z*-direction) to print the structure in a layer-by-layer or layerless continuous manner.^33,37,38^ Unfortunately, many DN resins do not meet the criteria of low viscosity and rapid photo-cross-linking at specific wavelengths. For instance, thermoreversible sol–gel transitions require elevated temperatures beyond the operating range of current printers, while reaction durations and cross-linking times for orthogonal click chemistries remain too long for PSLA (hours).^14,39^ New strategies that combine PSLA technology with DN hydrogels could potentially lead novel soft devices with superior mechanical properties.

Here, we report a simple one-pot PSLA printing strategy that combines the advantages of PSLA (rapid, high-resolution, 3D design flexibility) and hydrogels (transparency, hydration) to shape DN gels into complex geometries with superior mechanical properties (high strength in both tension and compression). Our methodology involved using acrylamide and κ-carrageenan as model monomers to construct the first and second networks, respectively. The photochemical cross-linking of the polyacrylamide, referred to as the first network, was initiated by the absorption of light by an LAP (light-absorbing photoinitiator) in the presence of a cross-linker. Simultaneously, the κ-carrageenan polymer underwent a coil–helix transition and the accumulation of double helices upon cooling, resulting in the formation of the physically cross-linked second network. The interpenetration of the κ-carrageenan and polyacrylamide networks led to enhanced mechanical properties.^40^ To demonstrate how this strategy can be used to make multifunctional soft devices, we printed an axicon lens using the DN gel and showed that dynamic stretching can be used to modulate its optical performance. This work can potentially expand the design freedoms and the material library for making stimuli-responsive soft devices using DN hydrogels.

## Methods

### Chemicals

All chemicals were used as received and were of analytical grade. Acrylamide (AAm), κ-carrageenan, *N*,*N*′-methylenebisacrylamide (MBAA), and tartrazine were purchased from Sigma-Aldrich. LAP was synthesized in our laboratory. Lithium phenyl-2,4,6-trimethyl-benzoyl phosphinate (LAP) was synthesized using a recognized method.^41^ Briefly, 2.85 mL of dimethyl phenylphosphonite was allowed to react with 3.00 mL of 2,4,6-trimethylbenzoyl chloride in a covered flask for 18 hours under a nitrogen blanket with stirring. After 18 hours, 100 mL of 6.25% (w/v) lithium bromide in 2-butanone solution was added to the flask. The mixture was heated to 50 °C and then allowed to react for 10 min. After cooling to room temperature, the white precipitate was collected via vacuum filtration and washed with four 100 mL aliquots of 2-butanone. Isolated LAP was dried in a vacuum for one week to remove the residual solvent.

### TOPS-Based 3D Printing of DN Hydrogels

**Figure 1:**
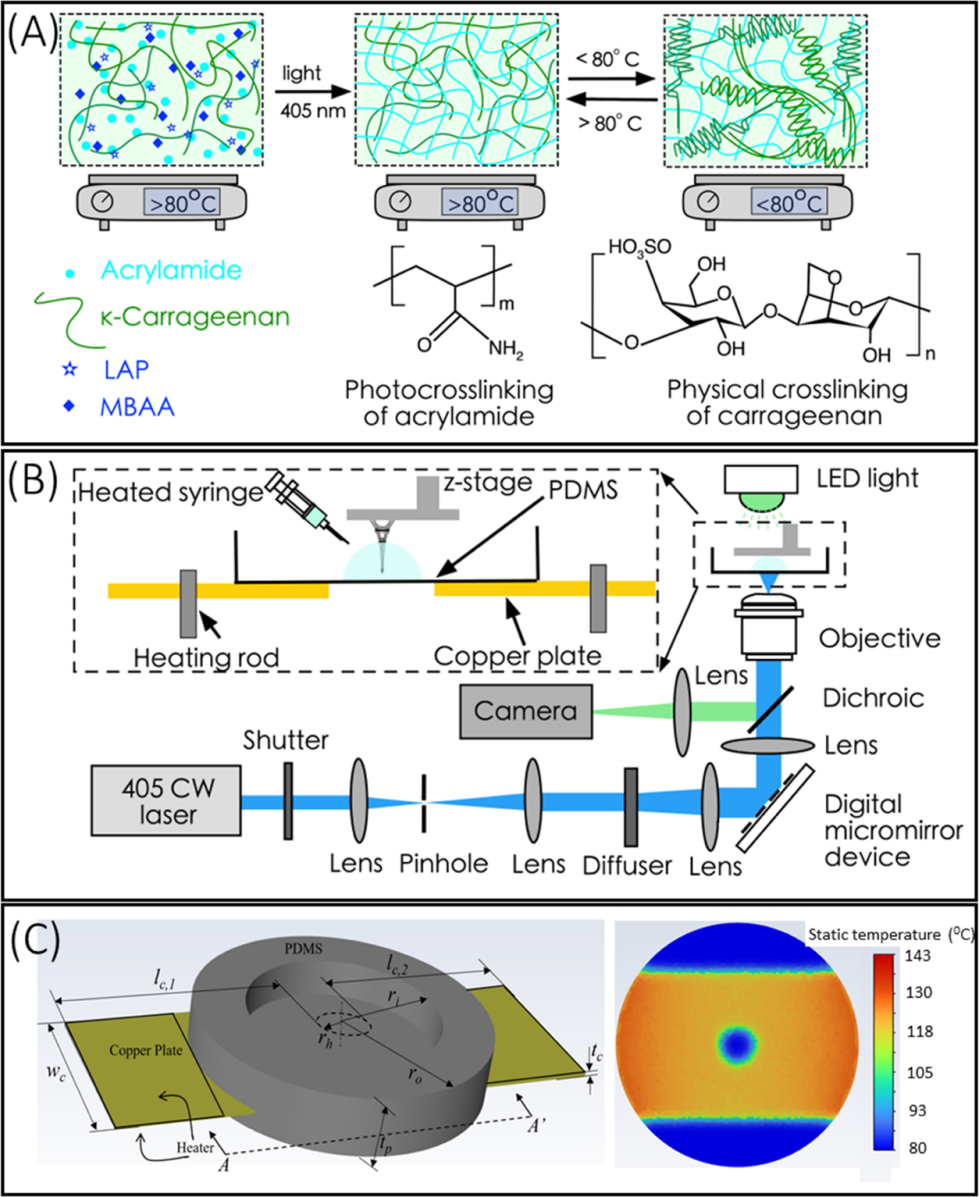
(A) Schematic diagram of DN gels formed by photo-cross-linking and thermoreversible sol–gel transition. Acrylamide in the presence of the cross-linker MBAA and photoinitiator LAP formed the first network through irreversible photo-cross-linking. κ-Carrageenan formed the second network by reversible physical cross-linking via sol–gel transition. (B) Schematic setup of the TOPS hydrogel for printing 2D and 3D DN gel structures. (C) The 3D model of the sample holder was used as a computational domain consisting of a PDMS dish with a copper plate. Temperature distribution over the PDMS layer at the plane corresponding to section A-A′ at *t* = 90 seconds when the steady state was reached.

A TOPS lithography optical setup used to fabricate the acrylamide/κ-carrageenan structures is shown (Figure 1). This setup consisted of a 405 nm CW laser (Toptica), which was expanded and spatially cleaned using a lens telescope and pinhole. The beam was directed toward the rotating diffuser, which changed the Gaussian intensity distribution of the laser beam to uniform intensity distribution. Rotation of the diffuser was used to average out the laser speckle. The diffuser diverged the laser beam, which was collimated using a lens and then the beam was projected into the digital micromirror device (DMD). DMD is a two-dimensional array of few micron-sized mirrors that spatially pattern the laser beam, which was directed toward the projection optics by a dichroic mirror. The projection lens assembly consisted of two lenses (*f* = 200 mm), which projected the beam with unity magnification into the prepolymer solution. An imaging arm was incorporated into the setup to monitor the fabrication process. The sample holder was fabricated with a copper plate and PDMS and is shown in the SI (Figure S1). A circular disk-shaped PDMS thin film was molded, and a copper plate with a circular hole of diameter 16 mm was thermally cross-linked on top of the PDMS disk. The rim of the sample holder was prepared by molding and casting and was thermally cross-linked on the top of the copper plate. The sample holder was heated using two heating rods. The presence of a circular hole in the copper plate imposes a size constraint on the printed parts in the *XY* dimension, limiting them to a maximum size of 16 mm. A linear actuator (PI) and a controller (G910, PI) were used to control the L-shaped *z*-stage. A LabVIEW program was written to coordinate the switch of the DMD masks, turning the laser ON and OFF, and the z-direction movement of the stage.

### Fabrication and Characterization of the DN Gel Structure

Fabrication was performed at an elevated temperature of 80 °C. First, a custom-written MATLAB code was used to slice a 3D model of interest and was used to create a stack of digital masks, which are binary portable network graphics (png) image files. The digital masks were uploaded to DMD, which selectively turn on/off the mirror to pattern the laser beam. This cross-linked a single layer inside the prepolymer solution. The change of the mask in the DMD was coordinated with the z-direction movement of the stage with a custom-written LabVIEW program. The laser beam photopolymerized the acrylamide to fabricate three-dimensional, transparent structures. Next, the structure was placed into an ice bath or cooled at room temperature to complete physical cross-linking.

### Digital Optical Microscopy Imaging

A digital optical microscope (HIROX, KH-8700) was used to image and characterize the printed structure with a resolution of 1.16 μm using an MX(G)-2016(z) objective lens.

### Tensile Testing

The tensile testing was performed using a tensile tester (250 lbs Actuator, Test Resources) at room temperature. Samples were printed into dog-bone shapes with a length of 6.25 mm and a gauge width of 1.50 mm. These structures were pulled at a rate of 150% strain (9.375 mm/min) using a load cell of 25 N. The modulus of elasticity was calculated as the maximum slope at the elastic region of the stress–strain plot. The fracture energy was estimated as the area under the stress–strain curve.

### Compression Testing

The compression testing was performed using a universal testing system (Model-5966, Instron) at room temperature. Cylindrical stud structures (radius = 7 mm, height = 5 mm) were printed and compressed at a rate of 0.5 mm/min.

### FTIR Studies

Infrared analyses were performed in attenuated total reflection mode (ATR) using a Bruker Tensor 27 FTIR spectrometer equipped with a MIRacle ZnSe single reflection ATR block and KBr beam splitter. Spectra of the printed dual network (DN) gel samples were recorded in the range 600–4000 cm^–1^ with a resolution of 4 cm^– 1^ and a sampling frequency of 32 scans. Samples were analyzed at three levels of hydration, both before and after tensile loading. Interfering peaks from water were removed from hydrated DN gel samples via subtraction of a spectrum averaged from replicate samples (n = 9) of Nanopure deionized water (18 MΩ·cm).

### Thermogravimetric Analysis

Thermogravimetric analysis (TGA) was performed on fully dehydrated DN gel samples using a TA Instruments Hi-Res TGA 2950 (TA Instruments, Waters Corporation, Milford, MA) in a nitrogen atmosphere between 25 and 600 °C with a heating rate of 10 °C/min. The thermal decomposition temperature was calculated at the onset of mass loss. The peak rate of mass loss was calculated using the relative maxima on a first derivative plot of weight percent vs temperature.

### Lens Stretcher

A custom “lens stretcher” was designed to stretch the printed optical constructs by desired increments. The stretcher was designed in Autodesk Inventor and assembled thereafter. The base plate was 3D-printed and designed to affix to a Thorlabs optical fixture. This allowed the entire stretcher to be mounted in line with the light path. The 3D-printed axicon lens is placed on the raised center platform, and the central hole in the base plate allows light to pass through both the plate and the lens. Four servo motors with linear actuation gearing were used to achieve uniform stretching of the constructs from all four sides. The servo motors were programmed via Arduino to move uniformly to any desired position in their range of motion. Small binder clips were fastened to the linear slides so the constructs could be attached firmly without slipping or tearing.

## Results

### TOPS Design and Printing of DN Hydrogels

Single-pot DN hydrogel prepolymer solution consists of acrylamide monomers, κ-carrageenan, cross-linker MBAA, LAP photoinitiator, and in some cases a photoabsorber. The basic mechanism of DN formation involves photo-cross-linking of the acrylamide network and physical cross-linking of κ-carrageenan below its sol–gel transition temperature (80 °C) (Figure 1A).^40,42−44^ Thus, a new stage was designed and built to allow the printing of the prepolymer formulation above 80 °C. Below 80 °C, prepolymer physically cross-links into a viscous gel and cannot be printed using PSLA. TOPS setup consists of a light source, diffuser, DMD, projection optics, z-stage, and a specifically designed sample holder for maintaining a constant elevated temperature (Figure 1B). The design of the sample holder consisted of a copper plate with a center hole embedded inside the PDMS bath. The 16 mm hole serves as the fabrication window, and the copper plate heats the sample holder. A multistep molding and casting technique were used to build the sample holder.

Computational fluid dynamics (CFD) simulation provided the temperature distribution to guide the design of the sample holder (Figures 1C and S1–S3). The temperature within the fabrication window was experimentally measured to be 80 °C when the heaters were operated at 150 °C. DN hydrogel structures were printed using PSLA at 80 °C as explained below. Based on user-defined CAD design, spatially modulated light patterns are irradiated onto the liquid prepolymer solution maintained at an elevated temperature. Upon irradiation, the LAP photoinitiator absorbs light and initiates a polymerization reaction in the presence of a cross-linker (MBAA) to form a polyacrylamide network that locks in place target geometry followed by cooling-driven physical cross-linking of the κ-carrageenan network, resulting in a DN hydrogel structure. For each layer, prepolymer solution was injected via a heated syringe into the fabrication window area, and post-printed samples were developed in hot DI water (at 80 °C) for 2 min to remove any uncross-linked monomers.

### Printing 2D, 3D Solid, and 3D Hollow DN Structures at Microscale Resolution

**Figure 2:**
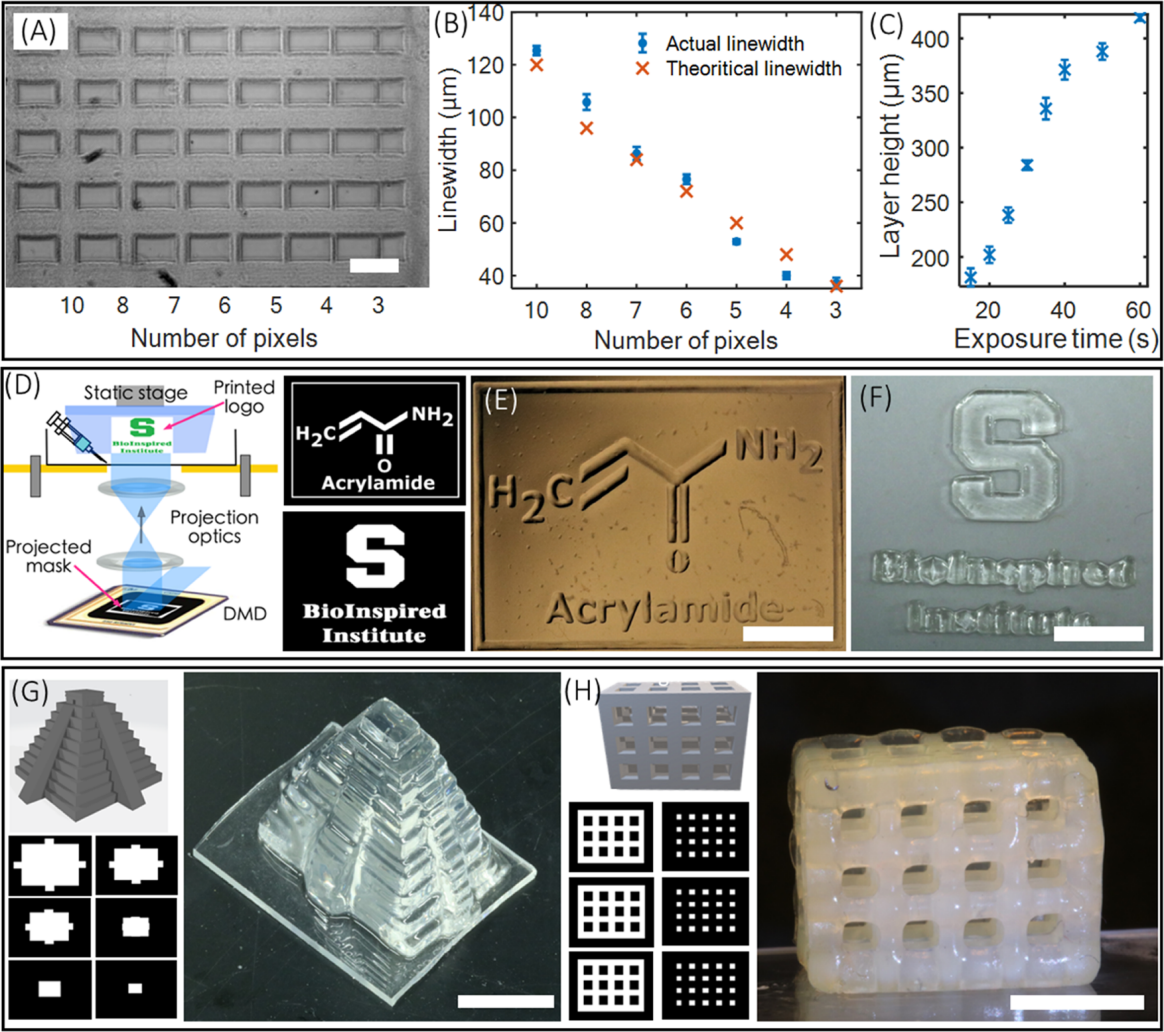
(A, B) Figure and plot depicting the lateral resolution of printed structure using DN gels (scale bar—200 μm). (C) Plot showing the axial resolution of the printed DN gel structure. (D) Schematic of 2D printing of the planar structure and computer-generated digital mask for printing chemical structure of acrylamide and logo of the BioInspired Institute. (E, F) 2D-printed acrylamide structure before the development and logo of the BioInspired Institute after the development of structure (scale bar—4 mm). (G) 3D CAD model, corresponding computer-generated digital mask, and 3D-printed structure of a Mayan pyramid (scale bar—5 mm). (H) CAD model, digital masks, and 3D-printed lattice structure. The printed structure was dipped into ethanol for 30 minutes to remove tartrazine and enhance the contrast for imaging (scale bar—5 mm).

Before printing complex, 2D/3D structures using DN hydrogels, both lateral and vertical resolution limits were characterized. The prepolymer composition used for this study was acrylamide (16 wt %), κ-carrageenan (2 wt %), MBAA (0.03 wt %), LAP (0.12 wt %), and water (81.85 wt %). The composition was used throughout the experiment, unless otherwise specified. For quantifying the lateral resolution of TOPS, digital masks of intersecting line patterns with varying pixels (3–10) were printed using a laser intensity of 2.17 mW/cm^2^ and an exposure time of 15 s. Measured line widths were close to the theoretical resolution, which corresponds to the micromirror size in the digital micromirror device (DMD) chip in PSLA (Figure 2A,B). For instance, the smallest feature size of 37 μm was experimentally obtained for a 3-pixel line pattern, close to the theoretical resolution of 36 μm. The vertical printing resolution, necessary to generate structures with internal voids, was characterized by adding a photoabsorber (tartrazine, 0.006 wt %) to the prepolymer solution. The curing depth was optimized by varying the exposure dose, a function of light intensity and time. Here, we printed a rectangular slab on the coverslip by varying the exposure time while maintaining a constant laser intensity of 2.4 mW/cm^2^. The thickness of the structures was measured to obtain the curing depth and plotted as a function of exposure time (Figure 2C). A *z*-resolution of 180 μm was obtained for the exposure time of 15 s. The curing depth can be tuned from 180 to 420 μm by varying the exposure time from 15 to 60 s. Exposure time below 15 s did not result in cross-linking of DN gel structures.

Results show a 2D pattern of the chemical structure of “acrylamide” and the logo of the BioInspired Institute of Syracuse University (Figure 2D–F). Next, a solid 3D geometry in form of a Mayan pyramid was printed with a laser intensity of 2.17 mW/cm^2^ and a layer exposure time of 15 seconds per layer (Figure 2G). Lastly, hollow 3D geometry with overhangs and undercuts was chosen and printed (Figure 2H). Here, a 0.006 wt % tartrazine (photoabsorber) was added to the prepolymer solution before printing the geometry (2.4 mW/cm^2^, exposure time per layer = 15 s). All printed structures were developed in water (80 °C for 2 min) to remove uncross-linked monomers.

### Tensile Performance of Printed DN Gel Structures

**Figure 3:**
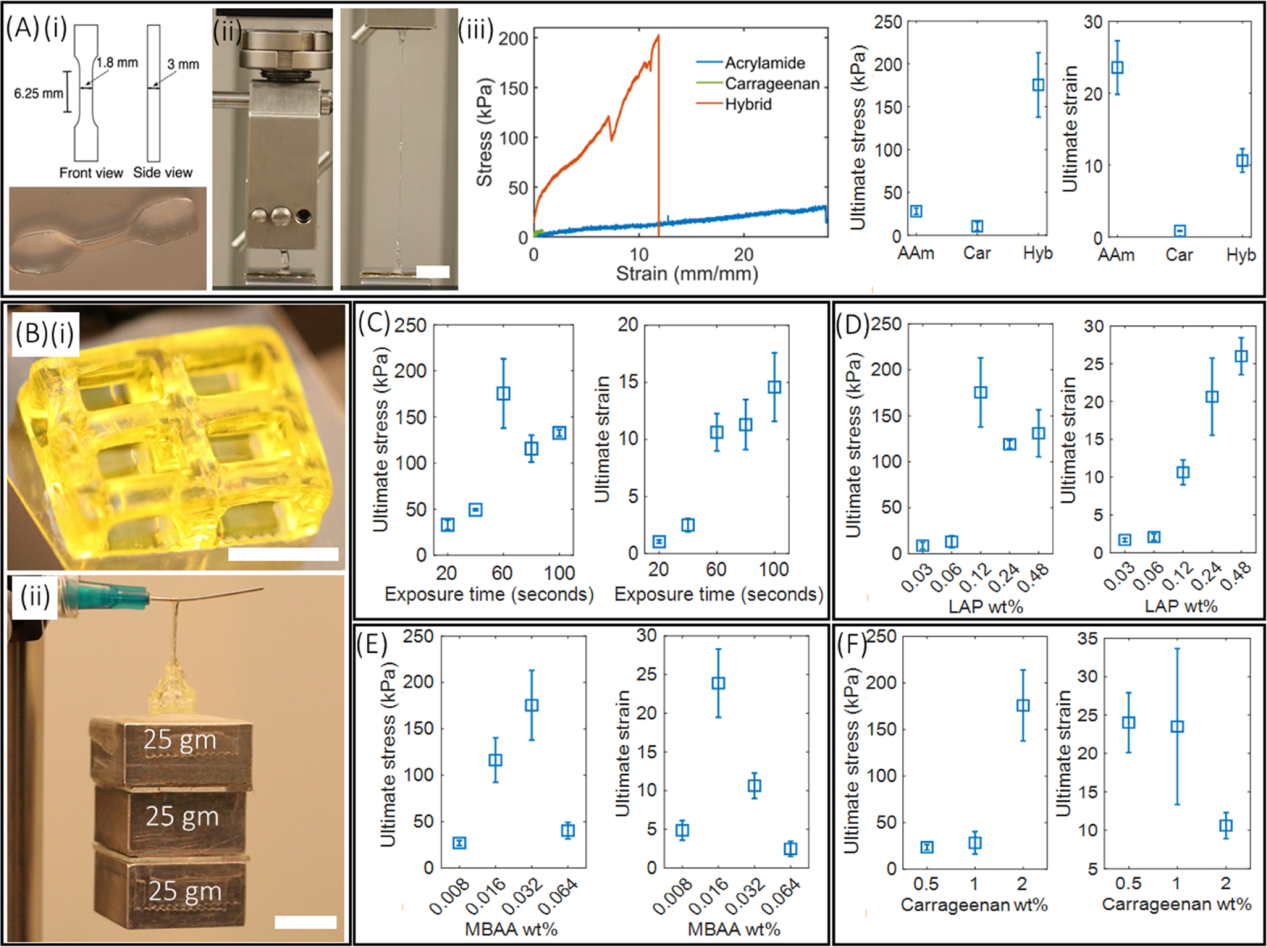
(A) (i) Schematic and printed dog-bone structure using a hybrid gel structure with a laser power of 2.17 mW/cm^2^ and an exposure time of 60 s. (ii) Photographs showing the performance of dog-bone structure during tensile testing, and the structure stretched 9.5 times its original length. (iii) Stress–strain plot obtained from dog-bone structures printed using the DN gel, acrylamide-only gel, and carrageenan-only gel. Ultimate stress and ultimate strain of the fabricated structures are also depicted. (B) (i) 3D-printed hollow-lattice geometry with a structure for studying the tensile performance (scale bar—5 mm). (ii) Photographs showing the tensile performance of the structure (scale bar—8 mm). (C–F) Ultimate stress and ultimate strain of DN gel structures printed by varying exposure times, and amounts of the photoinitiator, cross-linker (MBAA), and κ-carrageenan.

Tensile properties of TOPS-printed DN dog-bone-shaped structures (Figure 3A(i) and Video V1) were compared with identical structures made from single-network structures (acrylamide-only, κ-carrageenan-only). Acrylamide-only dog-bone structures were printed with TOPS (2.17 mW/cm^2^) using single exposure, while conventional molding and casting was used to generate dog-bone geometry using κ-carrageenan. The representative stress–strain plot shows the superior fracture energy (1238.1 J/m^2^) of DN structures compared to single-networked acrylamide (425.9 J/m^2^) and κ-carrageenan (4.25 J/m^2^) structures (Figure 3A(iii)). The ultimate stress required to stretch the hybrid gel structure by 10.6 ± 1.64 times was 175 ± 37 kPa, whereas the stress and associated ultimate strain for acrylamide gels were 28 ± 3.6 and 23.5 ± 3.7 kPa, respectively (Figure 3A(iii)). The κ-carrageenan structure breaks at the strain of 0.8 ± 0.06 and at the stress of 10.5 ± 4.9 kPa (Figure 3A(iii)). The modulus of elasticity is 79.5 ± 22 kPa, highest for the hybrid gel, whereas it was 8.2 ± 0.72 kPa for acrylamide and 7 ± 1.4 kPa for κ-carrageenan. Next, hollow-lattice geometry with a strut width of 900 μm was printed at 2.4 mW/cm^2^ with an exposure time of 15 s per layer (Figure 3B(i)). This structure can withstand a load of 75 grams for 20 s by stretching 8 times its original length (Figure 3B(ii) and Video V2). Overall, these results indicate that the hybrid gel structure obtains its strain from the polyacrylamide polymer, and the addition of κ-carrageenan increases the stiffness properties. It is suggested that a double helical structure in the κ-carrageenan starts to break at a small strain that unzipped progressively as the strain increases and leads to permanent deformation. The breaking of cross-linked double helices of κ-carrageenan serves as a sacrificial bond, which greatly helps to improve the stiffness of DN hydrogel through energy dissipation.^42^ Throughout the process, the acrylamide network remains intact and maintains the geometry of the printed structure. Standard dog-bone geometry was used to characterize the influence of many processing variables on tensile regimes.

### Influence of DN Formulations on Tensile Properties

#### Role of Light Dosage

Dog-bone DN gel structures were printed using TOPS under single-exposure conditions (in this case, the *z*-stage does not move) by varying the exposure times from 20 to 100 s at a constant laser intensity of 2.17 mW/cm^2^. Prepolymer composition used for this study was acrylamide (16 wt %), κ-carrageenan (2 wt %), MBAA (0.03 wt %), LAP (0.12 wt %), and water (81.85 wt %). Structures printed using the exposure time of 40 seconds and below broke with a very small stress of 50 kPa and exhibited a strain of less than 3 (Figure 3C). The structures printed with exposure times of 20 and 40 s were underexposed, and the associated covalent network of acrylamide is partially formed so that they exhibit less stretchability and accompanying stress. The highest ultimate stress of 175 ± 37 kPa was found for the exposure time of 60 s. Above this exposure time of 60 s, there is a slight decrease in ultimate stress, but the difference is not significant (Figure 3C). Similarly, the difference in strain is also minimum among the structure printed above 60 s although the highest ultimate strain of 14.59 ± 3 is observed for the structures printed with the longest exposure time of 100 s (Figure 3C). Further, the elastic modulus remains almost the same for the structures printed with different exposure times (Figure S4). Representative stress–strain plots obtained from the hybrid gel structures irradiated with different exposure times, and the corresponding elastic modulus obtained is depicted in the SI (Figure S4).

#### Role of Photoinitiator Concentration

Dog-bone DN samples were fabricated at a laser intensity of 2.17 mW/cm^2^ and an exposure time of 60 s using varying concentrations of LAP (0.03–0.48 wt %). The composition of acrylamide (16 wt %), κ-carrageenan (2 wt %), and MBAA (0.03 wt %) remained unchanged. Structures with 0.03 and 0.06 wt % LAP did not perform well while stretching and exhibited ultimate stress of less than 13 kPa (Figure 3D). The stress increased to 175.3 ± 37 kPa for the structure printed with 0.12 wt % LAP. Further, an increase in the concentration of the photoinitiator to 0.24 and 0.48 wt % decreased the ultimate stress to 119 ± 4 and 131 ± 25 kPa (Figure S5). There was an increasing trend in terms of ultimate strain and the maximum strain of 25.99 ± 2 kPa for printed structures with the highest LAP concentration (Figure 3D). Representative stress–strain plots obtained from the hybrid gel structures printed by varying photoinitiator concentration and corresponding variation in the modulus of the structure are reported in the SI (Figure S5). The modulus reaches a maximum of 79 ± 22 kPa for 0.12 wt % and dropped to 28 ± 5.4 and 33.8 ± 4.6 kPa for 0.24 and 0.48 wt %, respectively (Figure S5).

#### Role of Cross-Linker Concentration

Dog-bone DN samples were printed using TOPS with varying concentrations of MBAA (0.008–0.128 wt %), whereas the composition of acrylamide (16 wt %), κ-carrageenan (2 wt %), and LAP (0.12 wt %) was kept constant. Results showed that the highest ultimate stress of 175 ± 37 kPa and the ultimate strain of 10 ± 1.64 kPa were observed for 0.032 wt % MBAA. The ultimate stress decreased to 116 ± 24 kPa, while the ultimate strain increased to 23.8 ± 4.4 for the MBAA concentration of 0.016 wt % (Figure 3E). These parameters decreased significantly when the MBAA concentration decreased to 0.008 wt %. Furthermore, an increase in the concentration of the MBAA cross-linker to 0.064 and 0.128 wt % decreased both the stretchability and force required to break the dog-bone structure. The structure printed using an MBAA concentration of 0.128 wt % readily broke and hence was omitted for further studies (Figure 3E). The elastic modulus of 79.5 ± 22 kPa was the highest for an MBAA concentration of 0.032 wt % and the moduli were less than 30 kPa for structures printed with the other MBAA concentrations (Figure S6). Results show that an increase in the concentration of the cross-linker increases the cross-linking density, which leads to a short polyacrylamide chain and low fracture energy. For very high or low MBAA concentrations, the covalent network becomes too complaint, leading to deformation of the network with small stress.

#### Role of κ-Carrageenan Concentration

Dog-bone DN samples were printed with three concentrations of κ-carrageenan: 0.5, 1, and 2 wt % as above this concentration, κ-carrageenan did not dissolve in water. Herein, the composition of acrylamide (16 wt %), MBAA (0.03 wt %), and LAP (0.12 wt %) remained unchanged. As expected, the lower concentration κ-carrageenan structures were soft and highly stretchable. The strain decreased from 24 ± 3.9 to 10.6 ± 1.69, when the concentration of κ-carrageenan increased from 0.5 to 2 wt %, whereas the ultimate stress increased from 23.5 ± 3 to 175 ± 3 kPa (Figure 3F). The elastic modulus of 79.5 ± 22 kPa was the highest for the structure printed with 2 wt % κ-carrageenan and decreased with decreasing κ-carrageenan concentration (Figure S7). This result suggests that the κ-carrageenan increases the stiffness properties, whereas the acrylamide contributes to the stretching properties of the structure printed with hybrid gels.

#### Necking and Solution to Necking Phenomena

**Figure 4:**
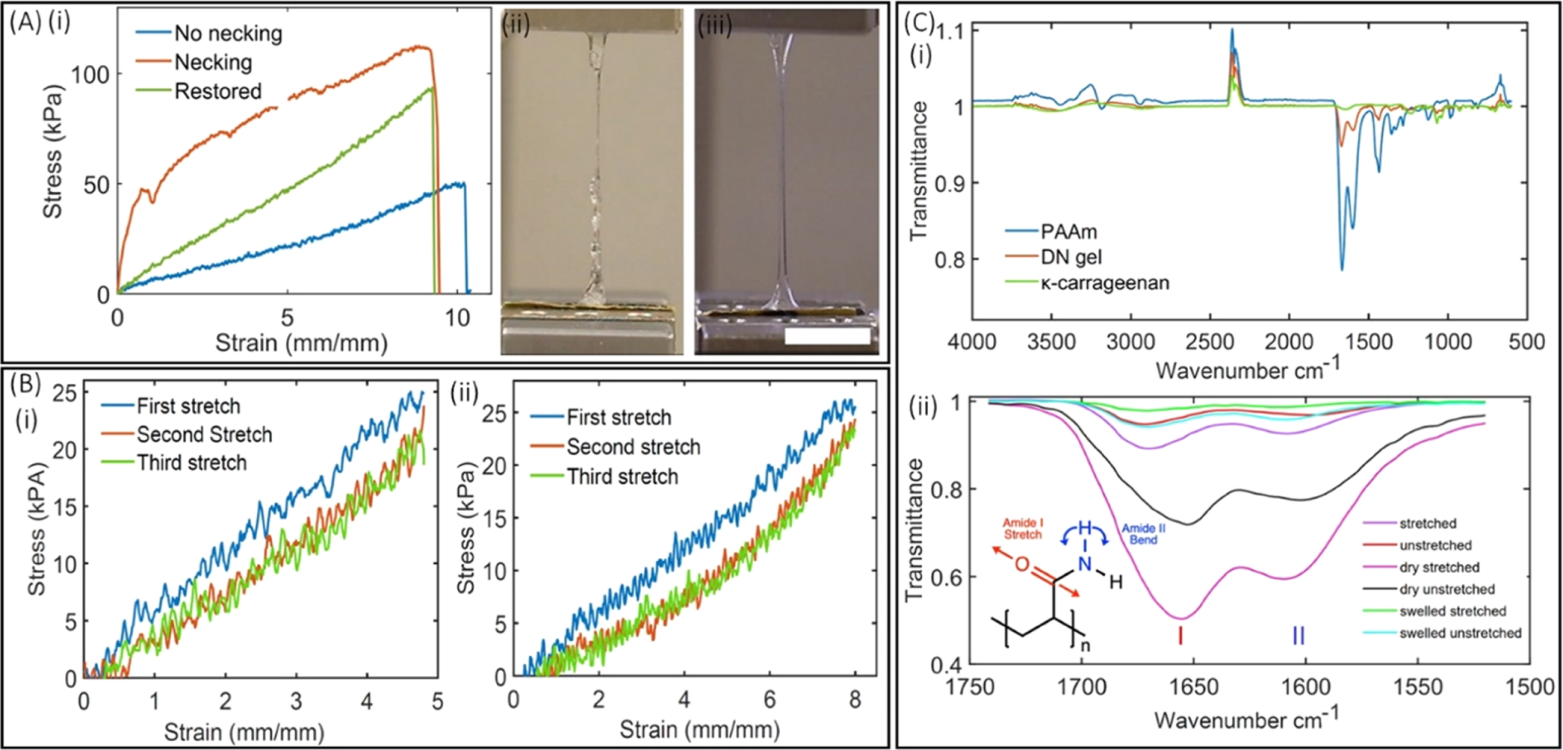
(A) Solution to the necking behavior: (i) comparison of the stress–strain plot of the structure with necking behavior (red), without necking behavior (blue), and without necking with restored stress (green); (ii, iii) photographs showing the necked and un-necked structure during stretching. (B) Stress plotted during three cycles of loading and reloading: (i) with the strain of (480%); (ii) with the strain of (800%). (C) (i) FTIR spectra of molded and cast κ-carrageenan, TOPS-printed PAAm, and DN gels; (ii) FTIR spectra, zoomed in, acquired from as-printed, dry, and swollen DN gel structures before and after stretching.

Many studies on DN hydrogels report a necking phenomenon at strain of ∼1.5 with variations in both their numbers and locations. Necking location corresponded to the point of breakage when more strain was applied. Furthermore, once necking is initiated, sample were not able to recover back to their original size (plastic deformation occurs) (Video V1 and Figure 4A). It has been proposed that necking occurs due to spatial variability within the material structure, which causes the material to experience disproportionate stress during deformation, leading to instability and resulting in localized strain in a specific area.^45^ We hypothesize that the cause of necking in printed DN samples is due to inhomogeneous spatial distribution of water within the structure, as the surface of the printed structure loses water faster than the center of the structure during the printing and post-processing steps. To test this hypothesis, we immersed printed DN samples in water for 5 minutes prior to recording stress–strain plots. Plots and the video show that the structures did not show the necking phenomenon (Figure 4A(i,iii) and Video V1) but exhibited lower stress compared to the as-printed structure possibly due to an increase in the total water content from 82 to 87%. To test this, another prepolymer solution was used to print the dog-bone sample with an initial (as-printed) water content of 60% and then immersed in water for 15 min to get the total water content to 82% before tensile testing. Results show a recovery of stiffness and associated stress with no necking behavior (green curve, depicted as restored in Figure 4A(i)). A detailed study of the swelling of the structure and the influence of swelling on the tensile properties of the structure is mentioned in the SI (Figures S8–S11).

### Response to Cyclic Loading–Unloading and FTIR Studies of DN Gels

#### Cyclic Loading–Unloading

DN dog-bones, hydrated for 10 minutes, were stretched to the strain of 480% for three repetitions, and fracture energy was calculated for all of the repetitions. Results showed that the fracture energy for the first cycle was 61.059 J/m^2^, which decreased to 44.92 J/m^2^ for the second cycle; however, the loss was only 0.94 J/m^2^ from the second to the third cycle (Figure 4B(i)). Similarly, stretching the structures to the strain of 800% for the three-repetition cycle showed a decrease in fracture energy from 98.67 to 69.41 J/m^2^ during the first and the second cycle while showing minimal energy loss of 1.93 J/m^2^ from the second to the third cycle (Figure 4B(ii)). Overall, energy loss increases with increasing strain (from 480 to 800%).

#### FTIR Studies of DN Gel Structures

FTIR spectra of cast κ-carrageenan, TOPS-printed PAAm, and DN gels (dried and swollen) are shown, and the FTIR fingerprints associated with functional groups are assigned (Figure 4C(i) and Table S2). FTIR spectra were acquired from as-printed, dry, and swollen dog-bone DN gel structures before and after tensile loading. The dry samples were obtained by drying them overnight at room temperature, and the swollen samples were obtained by immersing them in water for 4 hours. Of particular interest in this study are bands corresponding to moieties that participate in hydrogen bonds, such as the amide I (C=O stretching) band and amide II (N—H bending) band (Figure 4C(ii)). The observed FTIR bands and their assignments are summarized in Table 1. The amide I stretching band responds to the extent of hydration of the DN gel sample due to changes in the degree of hydrogen bonding.^46−48^ This band is shifted to a lower wavenumber in dried samples compared to hydrated, as-printed, and swollen samples due to an increase in intermolecular polymer–polymer hydrogen bonding. As evidenced by the absence of band shifting after tensile loading, the amide I band is relatively insensitive to changes in inter- and intramolecular forces induced by mechanical loading.^48^

**Table 1:** FTIR Fingerprints Associated with Functional Groups Amide I (C=O Stretching) Band and Amide II (N—H Bending) Band Obtained from Polyacrylamide, As-Printed, Dry, and Swollen Dog-Bone DN Gel Structures^a^

PAAm reported	PAAm	DN gel structure	DN gel structure (stretched)	dried DN gel structure	dried DN gel structure (stretched)	swollen DN gel structure	swollen DN gel structure (stretched)	assignment
1618	1600	1598	1608	1602	1610	1609	1609	δ, NH_2_ amide II
1660	1668	1672	1670	1653	1655	1671	1671	ν, C=O amide I

a ν Stretching; δ, bending.^48^

The amide II bending band is affected by tensile loading in as-printed and dried samples. Wavenumbers increase by 8–10 cm^–1^ to approximately 1609 cm^–1^ following tensile loading compared to the equivalent unloaded control observed at ∼1598 cm^–1^. This is indicative of a reduction in inter- and intramolecular interactions between polymer chains due to mechanically induced physical separation of the polymer chains. This can be attributed to the fracture energy loss during the structure’s cyclic loading (Figure 4B). Notably, no such mechanically induced band shift was observed for swollen samples, where the amide II band appears at 1609 cm^–1^ both before and after loading. The main reason for this phenomenon is the complete solvation of the polymer matrix with hydration shells forming around the polymer chains.^46^ The net result is a marked reduction in polymer–polymer interaction in the fully swollen state.^48^

#### Self-Healing Behavior

To assess if the thermoreversible sol–gel transition of the κ-carrageenan network can exhibit healing behavior at 80 °C, a pyramid-shaped structure was 3D-printed, cut into half using a sharp razor blade, and was stained with pink and blue dyes. The two pieces of the structure were sealed in a polyethylene bag and stored in a water bath of 85 °C for 20 min. The two halves of the structure self-healed to produce a complete pyramid (Figure S12A,B). To quantify the self-healing behavior, a rectangular slab geometry, printed using TOPS, was cut in half using a sharp razor blade. To visualize the self-healing interface, one of the halves was stained with faint blue dye. Then, the two halves were placed in close contact, heated to 80 °C for 20 min, and cooled down to initiate physical cross-linking of the κ-carrageenan network (Figure S12C,D). The self-healed monolithic structure was able to withstand a weight of 70 gm, although the self-healed interface starts to rupture when the load is increased to 200 gm (Figure S12E,F). Similar experiments with self-healed DN dog-bone samples resulted in an ultimate stress of 24 ± 3 kPa and breaks at a strain of 2.5 ± 0.6, much lower than those of as-printed samples (ultimate stress of 125 kPa and ultimate strain of 15.1) (Figure S12G). This is because self-healing takes place solely via physical cross-linking of the κ-carrageenan network.

To explain the self-healing behavior, thermogravimetric analysis of dehydrated DN gel samples (n = 4) was performed. Results showed a three-stage thermal decomposition (Figure S13). The onset of the first stage occurred at 224.10 ± 3.08 °C with a peak rate of mass loss at 236.03 ± 1.38 °C. The second stage had an onset of 263.99 ± 0.80 °C and reached a peak rate of mass loss at 279.83 ± 0.54 °C. The third stage had an onset of 360.37 ± 0.82 °C and reached a peak rate of mass loss at 382.04 ± 0.91 °C. The residue weight percent at 600 °C was 25.00 ± 0.75%. As shown, the onset of thermal decomposition is well in excess of the thermal processing conditions utilized in both printing DN gel structures and inducing self-healing of the physically cross-linked network. Though TGA was performed on dehydrated samples, these results are believed to be representative of the thermal stability of hydrated DN gel structures due to the absence of hydrolysis-sensitive linkages at neutral pH. The self-healing behavior exhibited by DN gels can therefore be ascribed to the interdiffusion of solvated κ-carrageenan chains and subsequent reformation of physical cross-links rather than decomposition into highly adhesive oligomers.

### Compressive Performance of DN Hydrogel Structures

**Figure 5:**
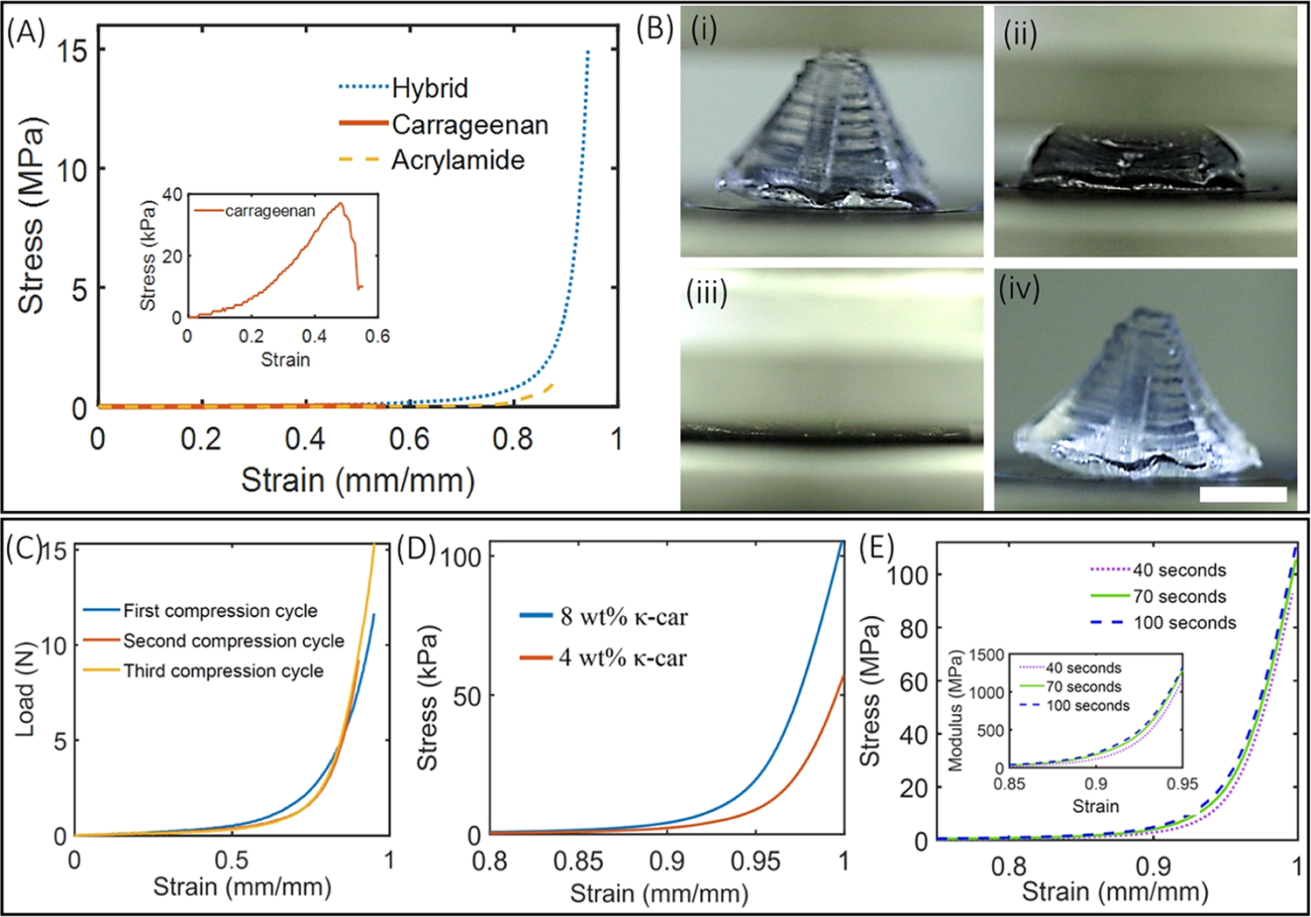
(A) Stress–strain plot obtained from the cylindrical stud structure printed using acrylamide-only, κ-carrageenan-only, and acrylamide/κ-carrageenan hybrid gels. Inset shows a strain–stress plot of the κ-carrageenan structure. (B) Demonstration of compression and recoverability of a 3D-printed Mayan pyramid structure (scale bar—5 mm). (C) Force–strain plot for 3 compression cycles of the pyramid structure. (D) Compressive stress–strain plot for structures printed using two different concentrations (4 and 8 wt %) of κ-carrageenan in the hybrid gel. (E) Stress–strain plot for DN gel structures printed by varying laser exposure time at fixed laser intensity. Inset shows the change in the modulus of structures printed using different exposure times.

Mechanical properties of DN cylindrical stub structures (radius = 7 mm and height = 5 mm) under compression were compared with identical structures made from single-network structures (acrylamide-only and κ-carrageenan-only). Cylindrical stubs, printed using 2.17 mW/cm^2^ and an exposure time of 70 seconds, were subjected to uniaxial compression, and associated stress and strains were plotted (Figure 5A). Results show that the ultimate compressive stress in the case of the DN structure was 15 MPa at a strain of 95%, which is 10- and 150-fold the stress possible by acrylamide and κ-carrageenan structures, respectively. κ-Carrageenan-only structures fracture at 0.032 MPa and a strain of 50%, while polyacrylamide-only structures fracture at 1.45 MPa and a strain of 89% (Figure 5A). The result suggests that both the κ-carrageenan network and acrylamide network increase the toughness of the hybrid structure not just by simple interpenetration but also through a possible synergistic interaction of two networks. Next, TOPS was used to print a 3D Mayan pyramid and tested under compression (Figure 5B and Video V3). Even after 95% strain for 3 cycles, the structure recovers back to its original shape with little deformation upon unloading. A small difference is observed in the force–strain plots between cycles 1 and 2, while little-to-no differences are observed between cycles 2 and 3, demonstrating superior shape recoverability of printed structures (Figure 5C). Compression tests were performed on the swelled structures, which were immersed in water for 4 days. The original structure swelled almost 5 times its volume, and the compression result showed that these structures can withstand the ultimate compression strain of 84% at a stress of 0.1 MPa, which is 150 times smaller than the ultimate stress associated with the original structure (Figure S14).

Specific roles of κ-carrageenan concentration and exposure times on the compressive properties of printed DN structures were also studied. Decreasing the concentration of κ-carrageenan decreases the ability of the structure to withstand loads, consistent with the trends seen in tensile testing (Figure 5D). Structures printed with lower exposure times were found to be softer compared to those printed using a longer exposure time (Figure 5E, inset). Longer exposure time strengthens the covalent bond of the acrylamide network, thereby increasing the stiffness of the material.

#### Shaping DN Hydrogels into a Dynamically Tunable Soft Photonic Device

**Figure 6:**
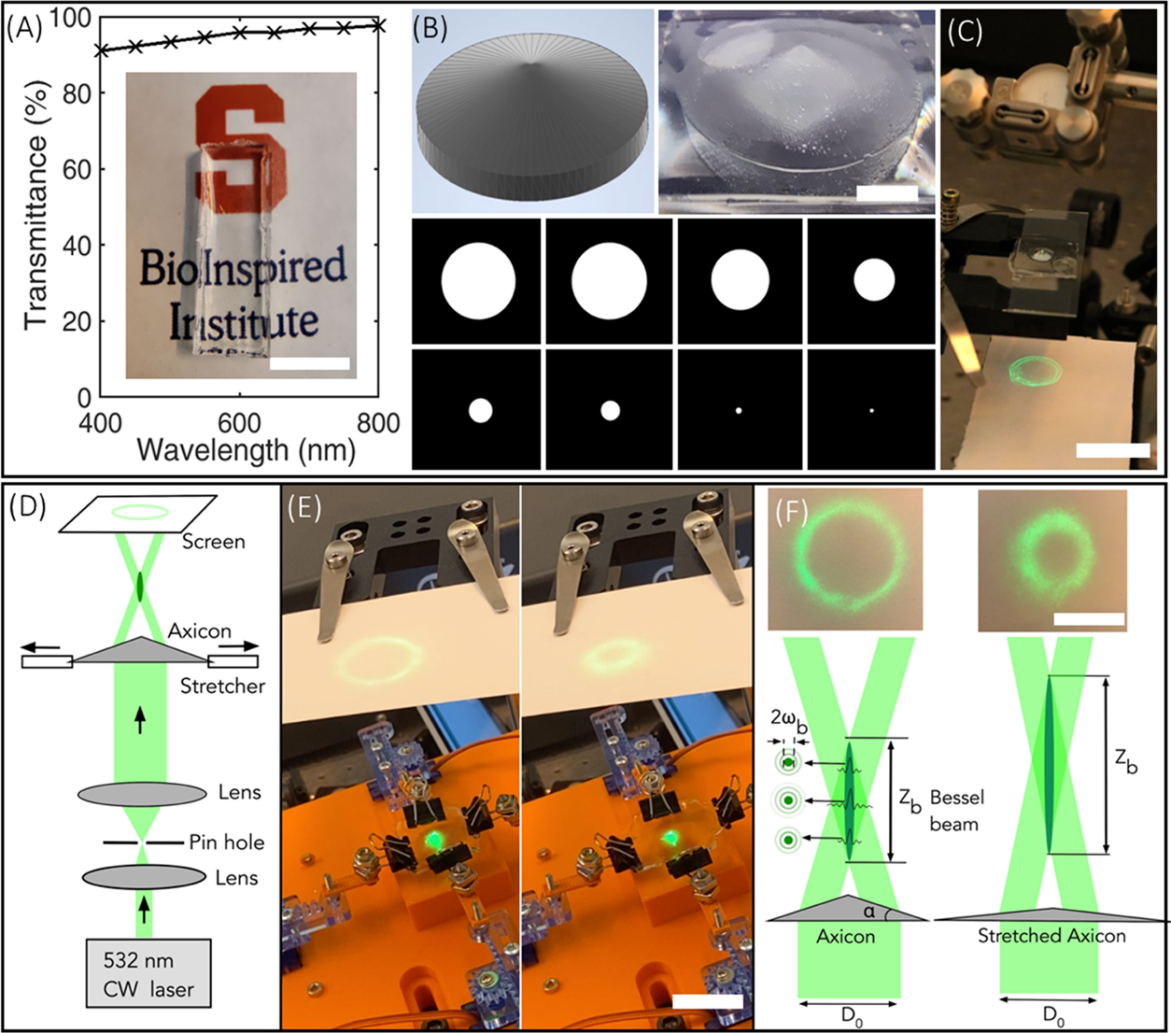
(A) Transmission spectra of the structure printed using the DN gel structure. The printed structure is placed on the top of the BioInspired Institute logo to demonstrate the high transmissivity of the DN gel structure (scale bar—1 cm). (B) CAD design, computer-generated digital masks, and 3D-printed axicon lens using TOPS (scale bar—2 mm). (C) Characterization of the annular ring while the axicon lens is static (scale bar—2 cm). (D) Optical setup to characterize the annular ring of the axicon lens during dynamic stretching. (E) A screenshot was obtained from Video V4 showing the tunability of the axicon lens (scale bar—3 cm). (F) Schematic showing the zero-order Bessel beam generation by the axicon and experimentally obtained annular rings before and after stretching the axicon lens (scale bar—2 cm). ω_b_ = radius of the central lobe, *z*_b_ = length of Bessel region, *D*_0_ = diameter of incident beam.

Here, we printed an axicon lens (a conical prism) capable of generating a dynamically reconfigurable quasi-Bessel beam and its characteristic annular ring. First, the transparency of the DN hydrogels was characterized. The transmission spectra of printed DN geometry showed a transmissivity of more than 90% over the wavelength of 400–800 nm (Figure 6A). A printed slab of DN gels structures clearly showed the logo of the BioInspired Institute, depicting the high transparency of the structure (Figure 6A, inset). Next, TOPS was used to print an axicon lens with a laser intensity of 2.17 mW/cm^2^, an exposure time per layer of 15 s, and a layer thickness of 50 μm (Figure 6B). The diameter and thickness of the as-printed lens are 8 and 3.65 mm, respectively, and the base angle (β) is measured to be 24.5°. In as-printed static conditions, a Gaussian beam passed through the axicon lens and generates an annular ring (Figure 6C). Then, biaxial tensile stress was applied to the DN axicon lens using a custom-built stretching device (Figure 6D,E). Dynamic stretching of the DN lens results in a corresponding increase in the cone apex angle and a decrease in the diameter of the annular ring as visualized using a digital SLR camera (Figure 6F and Video V4). Details of the optical setup and stretching devices are provided in the Methods section. Mechanically reconfigurable DN axicon lenses represent a new class of soft multifunctional photonic devices.

## Discussion

This work reports TOPS-enabled DN hydrogel structures that exhibit superior mechanical properties in both tensile and compression regimes using an optimized formulation composed of acrylamide (16 wt %), κ-carrageenan (2 wt %), MBAA (0.03 wt %), and photoinitiator LAP (0.12 wt %). Photo-cross-linking of the primary acrylamide network ensures the structural integrity during printing while cooling below the sol–gel transitions temperature of 80 °C results in physical cross-linking of the secondary κ-carrageenan network. Since the fracture energy of the DN structures is greater than the sum of fracture energies of individual network structures, it points to the synergistic effect of cross-linking and chain entanglements between the two networks. Tensile tests show that acrylamide and κ-carrageenan networks contribute to stretchability and stiffness, respectively. Results show that many processing variables modulate the mechanical properties of printed structures. For instance, the development of printed structures above 80 °C and a duration of more than 3 min can lead to heat-induced distortions of the DN structures. An increase in cross-linker (MBAA) concentration shows low fracture energy, while an increase in exposure times increases the stiffness of the material. Necking behavior, seen in as-printed samples, can be resolved by simply immersing the samples in DI water for a few minutes, which removes local defects induced by nonuniform hydration. Although possible, self-healed DN structures exhibit inferior ultimate stress and strain compared to the as-printed structures. The compression properties of these structures are comparable to the mechanical properties of bovine cartilage and surpass previously reported 3D-printed DN hydrogel structures, as explained below.

### Comparison of Mechanical Properties and Resolution: State-of-the-Art vs TOPS-Enabled DN Gel Structures

**Figure 7:**
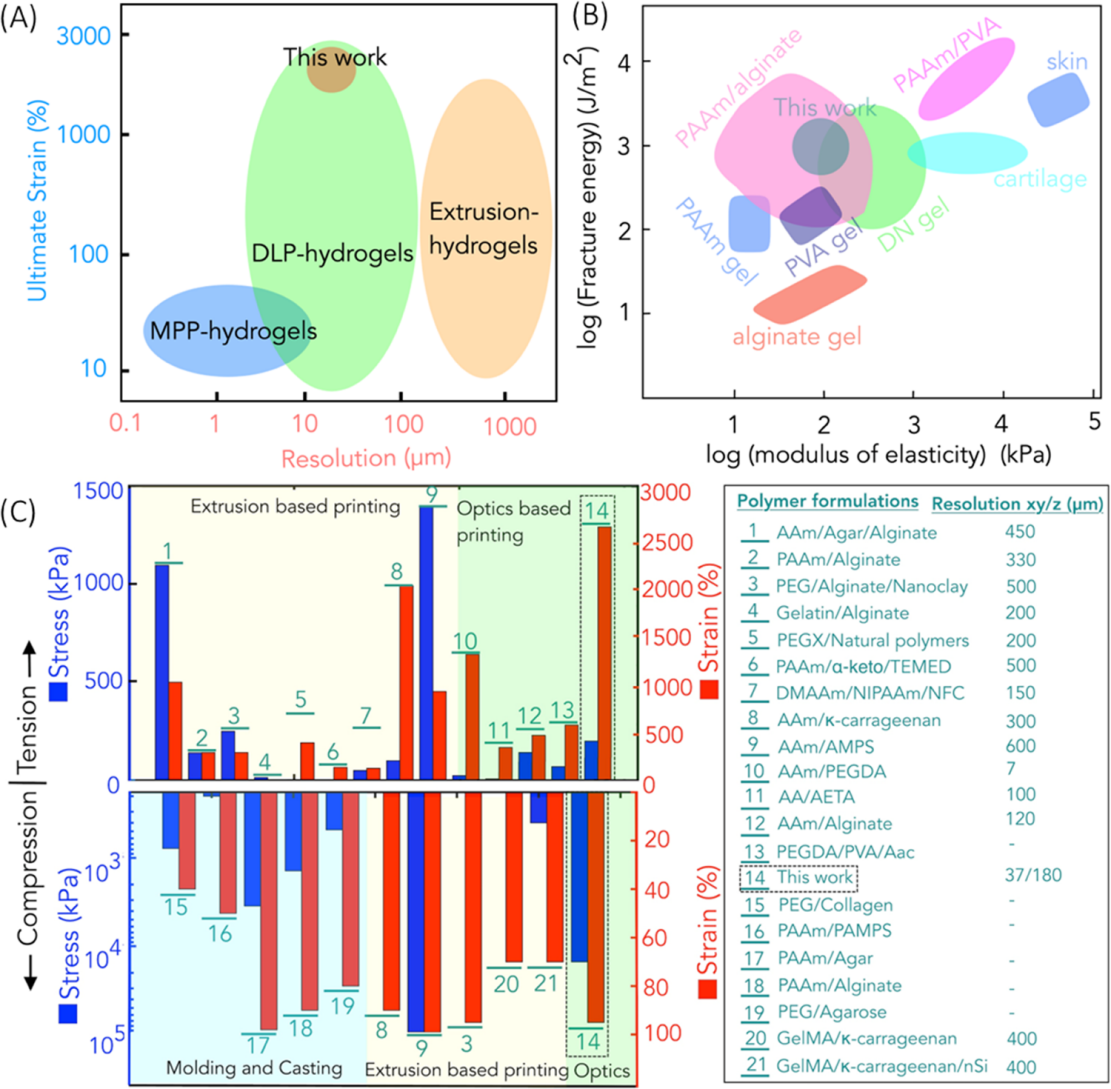
(A) Comparison of performance of TOPS-printed acrylamide/κ-carrageenan structures with existing technology of hydrogel fabrication in terms of lateral resolution and ultimate strain. (B) Comparison of performance of TOPS-printed acrylamide/κ-carrageenan structures with hydrogels and double-network hydrogels in terms of fracture energy and modulus of elasticity. (C) Plot depicting the mechanical performance (in terms of strain and strain) and comparison of printing performance (in terms of resolution) of TOPS-printed acrylamide/κ-carrageenan structures with other 3D-printed DN hydrogels. Note: all other studies only report lateral (*XY*) resolution. Since we printed 3D hollow structures with overhangs and undercuts, we have also reported the *z* (depth) resolution.^26,27,30−32,49,52,53,55−59^

Among various fabrication methods at our disposal, extrusion-based direct ink writing (DIW) is the most widely used; however, achieving a resolution of 200 μm or less remains challenging.^49^ Multiphoton polymerization-based 3D printing techniques can print high-resolution structures at a micrometer scale; however, they are extremely slow owing to their serial point-by-point scanning.^50,51^ The resolution of the TOPS lithography system is comparable to different kinds of optical projection lithography such as DLP or CLIP, which support the rapid fabrication of 3D hydrogel structures with a resolution of approximately 10–200 μm^33,35,38^ (Figure 7A). In contrast to other work, which focuses on one regime (tensile or compression), TOPS-printed 3D structures simultaneously exhibit superior mechanical properties in both tensile regimes (strain of 2400% and stress of 130 kPa) and compression regimes (strain of 95% and stress of 15 MPa) with a high degree of recoverability. Moreover, fracture energy (1238.1 J/m^2^) and modulus of elasticity (98 kPa) are comparable to most tough hydrogels, double-network hydrogels, and tough soft biotissues (Figure 7B).

As far as we know, this unique combination of high-resolution, 3D design flexibility, stretchability, compressibility, and recoverability is better than current state of the art, which includes DN structures printed using light and extrusion-based printing methods as well as conventional casting/molding strategies. Since the literature does not report all aspects, we tried our best to compare our work with the existing literature using a chart showing ultimate stress and strain in both tensile (upper part) and compression (lower part) regimes (Figure 7C). The best resolution was obtained for the optics-based printing of PAAm/PEGDA, which showed a resolution of ∼7 μm.^32^ The smallest feature size obtained for our work after the development stage was 37 μm. Structure as small as 12 μm was printed; however, they did not survive the development stage. The ultimate tensile stress of TOPS DN structures is comparable with other 3D-printed DN structures, whereas the ultimate strain of our samples is better than most other DN structures. Closest to our strain response in tension (2400%), extrusion-printed PAAM/κ-carrageenan showed a strain of ∼20,^26^ whereas the optics-based dual photo-cross-linking of AAm/PEGDA showed the ultimate strain of ∼12.^32^ Our compression strain (95%) is similar to the reported work, for instance, extrusion-based printing of PEG/alginate/nanoclay^52^ and molding/casting using agar/PAAm.^18^ The ultimate compression stress was highest for the AAm/AMPS, which is 93.5 MPa; however, it is not clear if the structure can recover after the compressive stress is removed.^53^ In our case, a printed structure with an ultimate stress of 15 MPa can recover. The only study with comparable mechanical properties was the Aam/AMPS; however, the resolution of printing of this material system is limited to 600 μm, while the resolution of this work is 37 μm.^53^ Based on this, TOPS-based DN structures exhibit better mechanical properties compared to other DN structures.

Further, the versatility of this single-step manufacturing process extends beyond the materials currently employed, allowing for temperature adjustment to accommodate other temperature-responsive physically cross-linking substances like agar, gelatin, and more. Additionally, this method enables the printing of highly viscous materials such as difunctional urethane dimethacrylate and triethyleneglycol dimethacrylate that exhibit a decrease in viscosity as the temperature rises.^54^

## Conclusions

TOPS allowed the printing of 3D structures while maintaining the desired temperature of the prepolymer solution. This optical technique of fabrication was demonstrated to print the DN hydrogel of acrylamide and κ-carrageenan at the resolution of 37 μm. The as-printed 2D/3D structures were complex, mechanically strong, highly stretchable, and transparent and exhibited tunable mechanical properties by varying the printing conditions, material formulations, and post-processing steps. The printed structures performed equally well under compression and tensile force, unlike most other 3D-printed DN gel structures, which only performed well under either tensile or compression force. As a proof of concept, a mechanically reconfigurable axicon lens was printed using TOPS, paving the way to print on-demand 3D elastomeric transparent structures, and can be utilized in a range of applications such as soft robotics, soft wearable electronics, adaptive optics, augmented reality, tissue engineering, and regenerative medicine.

## Supporting Information Available

Specifics related to materials and methods, computational domain consisting of PDMS dish connected with a copper plate, temperature distribution over the PDMS layer, temporal evolution of temperature distribution over the PDMS layer heated using the copper heater, stress–strain plots, image and plot of swelling of TOPS-printed DN gels, images of dog-bone structures after swelling and deswelling, self-healing properties of 3D-printed Mayan pyramid structure, TGA plot, CAD design before and after assembly of axicon lens stretching device, dimensions of geometry associated with the computational domain, assignment of relevant FTIR fingerprints associated with functional groups, experimental details, materials, methods, and results (PDF)
Stretching of TOPS-printed acrylamide/κ-carrageenan double-network hydrogel dog-bone structures (Movie V1) (MP4)
Stretching of TOPS-printed acrylamide/κ-carrageenan double-network hydrogel 3D structures (Movie V2) (MP4)
Compression of a TOPS-printed Mayan pyramid using the acrylamide/κ-carrageenan double-network hydrogel (Movie V3) (MP4)
Tunability of the axicon lens using a custom-designed lens stretcher (Movie V4) (MP4)
