# Supplementary material for: Printing Double-Network Tough Hydrogels Using Temperature-Controlled Projection Stereolithography (TOPS)
Source: ACS Appl Mater Interfaces. 2023 Jun 15;15(25):30780–92. doi: 10.1021/acsami.3c04661 (PMC10316326; doi:10.1021/acsami.3c04661)
Supplement: Supplementary file 1 — am3c04661_si_001.pdf [file am3c04661_si_001.pdf]

Supporting Information

Printing double network tough hydrogels using Temperature-Controlled Projection Stereolithography (TOPS)

*Puskal Kunwar[#@], Bianca Louise Andrada[#@], Arun Poudel[#@], Zheng Xiong[#@], Ujjwal Aryal[#@], Zachary J Geffert[#@], Sajag Poudel, Daniel Fougnier[#], Ivan Gitsov[$@], Pranav Soman[#@]**

[#] Syracuse University, Biomedical and Chemical Engineering Department,

[@]BioInspired Institute, Syracuse, New York, 13210, USA

[$] State University of New York ESF, Department of Chemistry, Syracuse, New York, 13210, USA; The Michael M. Szwarc Polymer Research Institute, Syracuse, New York, 13210, USA

Dimension of the sample holder

The dimensions associated with the geometry are provided in Table 1.

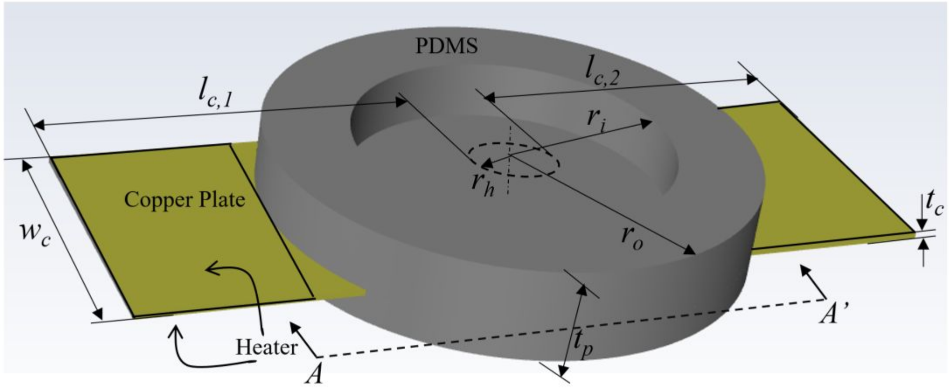

Figure S1. Computational Domain consisting of PDMS dish connected with copper plate.

Table S1. Dimensions of geometry associated with the computational domain are shown in Fig.1.

Symbol	w_c	$l_{c,1}$	$l_{c,2}$	r_i	r_o	r_h	t_p	t_c
Dimension (mm)	53	65	70	30	38	8	17.5	1.1

Heat Distribution Simulation

It was important to maintain a critical temperature of the solution throughout the fabrication process, hence we designed a CAD model of the sample holder and studied the temperature distribution using simulations. The design of the sample holder consisted of a copper plate with a center hole embedded inside the PDMS bath (**Figure S1**). Two heated rods on either side of the dish were designed to heat the copper plate and the 16mm diameter hole in the Cu plate acted as the fabrication window. To gain a better insight into the temperature distribution over the PDMS

layer of the sample holder design, we performed computational fluid dynamics (CFD) simulation with conjugate heat transfer. The computational domain consisted of the designed PDMS dish and a copper plate extended on either side of the dish (**Figure S1**). At the top and bottom surface of the copper plate, a constant temperature boundary condition with T = 416 K (142.85°C) was applied to mimic the heater (used in an experimental study). All other surfaces of the geometry were provided with convection heat transfer to ambient temperature (T_{amb} = 300 K (26.85°C).

The simulation was performed by discretizing the computational domain into a finite number of control volumes (or grid points) and by simultaneously solving the physical equations of continuity, momentum, and energy, in each point to obtain the spatial and temporal distribution of temperature. For the computational domain, a mesh with 160,000 grid points was utilized to obtain the temperature distribution (**Figure S2**). The distribution was obtained for various time points until the steady state is attained. After the simulation study, the mesh was refined, and the simulation was repeated for meshes with 200,000, 240,000, and 280,000 grid points to investigate the grid sensitivity of the result. By comparing temperature distribution over the PDMS layer for different meshes, one with 240,000 grid points was found to be an optimum mesh which is utilized for further study here.

Finite volume methods-based commercial solver ANSYS Fluent was utilized to solve the equations. In the simulation, the entire domain was first initialized with T_{init} = 353 K (79.85 °C), and the ambient was set at 300 K (26.85°C). The operating pressure was 1 atm. **Figure S3** shows the temperature distribution over the PDMS layer at different time instants obtained using the optimum mesh. At approximately t = 90 s, the spatial distribution of temperature attains a steady state and further continuing the simulation shows no changes in the state.

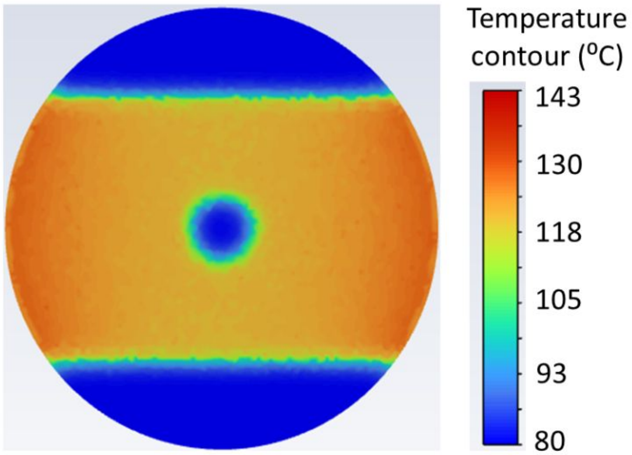

Figure S2. Temperature distribution over the PDMS layer at the plane corresponding to sections A-A' shown in Figure S1.

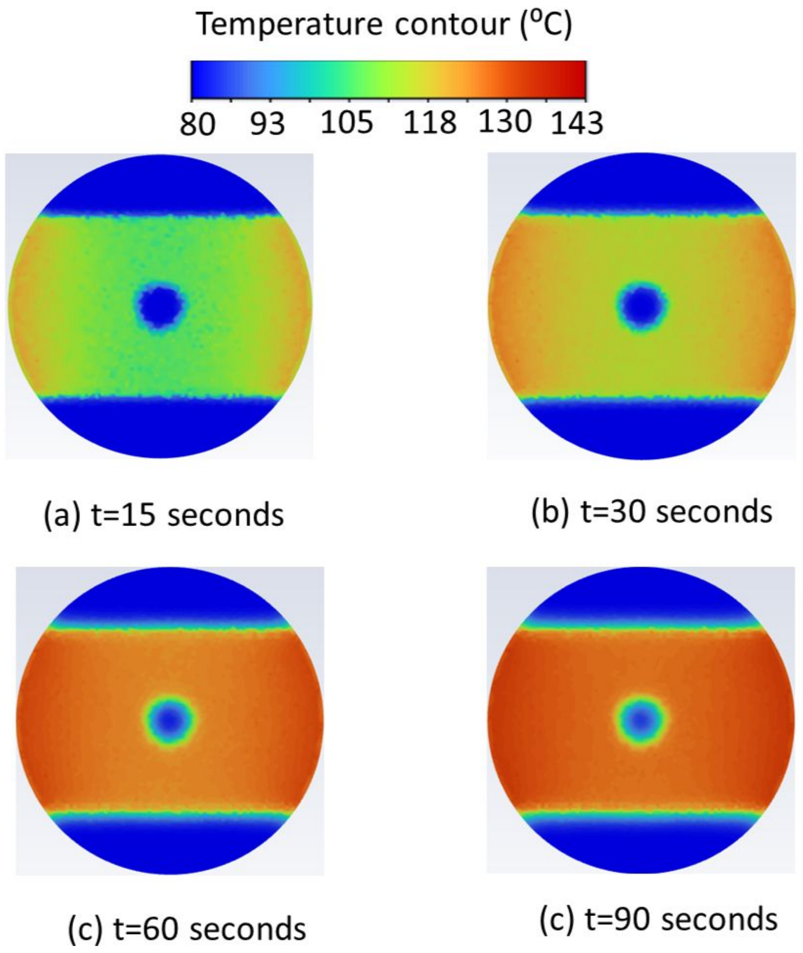

Figure S3. Temporal evolution of temperature distribution over the PDMS layer heated using the copper heater at different time points (a) 15 seconds, (b) 30 seconds, (c) 60 seconds, and (d) 90 seconds. The plots are obtained from the simulation using an optimum mesh of 240,000 grid points.

Effect of exposure time on the tensile properties of TOPS printed DN gel dog-bone structures

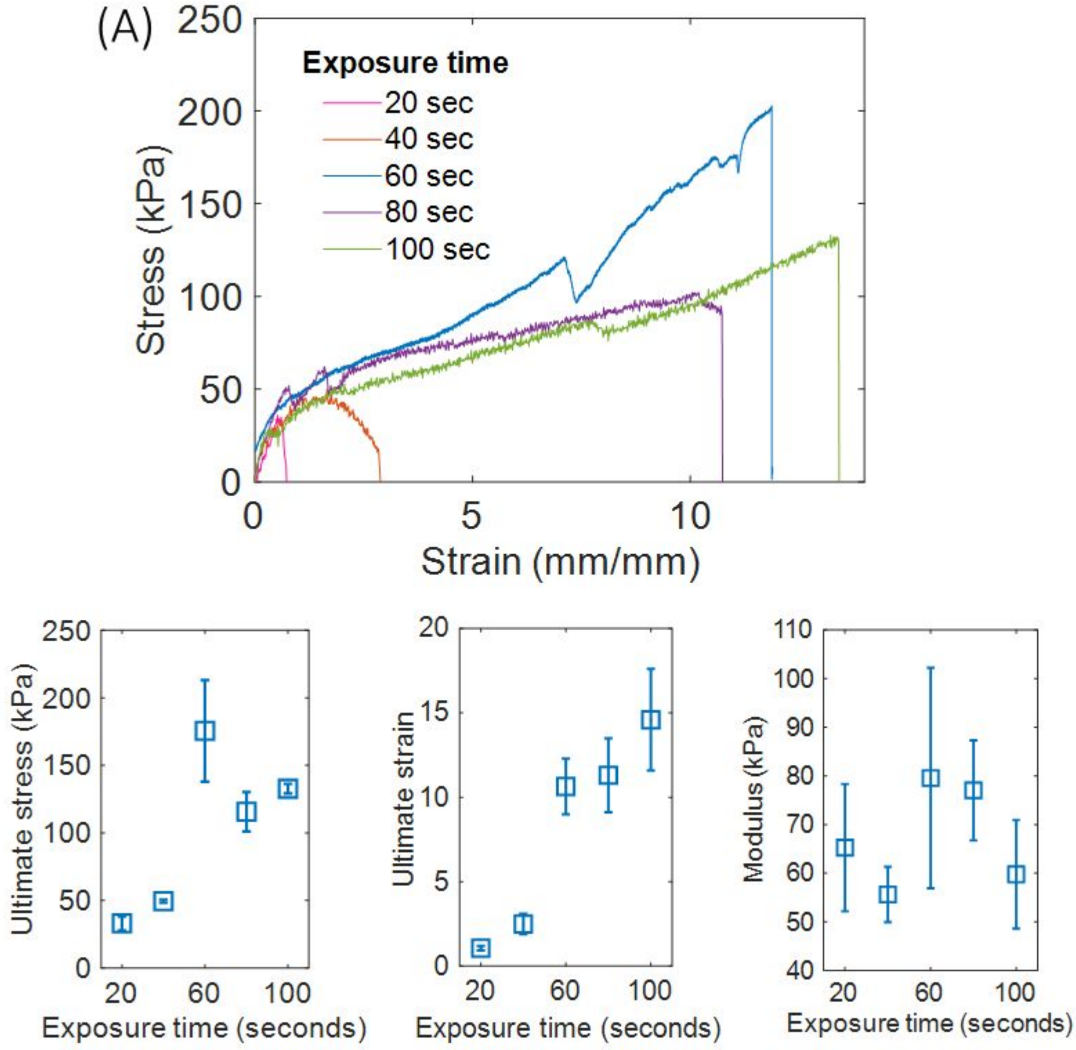

Figure S4. (A) Stress−strain plots obtained from TOPS printed DN gel structures exposed to different exposure times. Ultimate stress, ultimate strain, and tensile modulus recorded for the TOPS printed structures with varying exposure times. (Error bars: Mean±SD)

Effect of composition of photoinitiator on the tensile properties of TOPS printed DN gel dog-bone structures

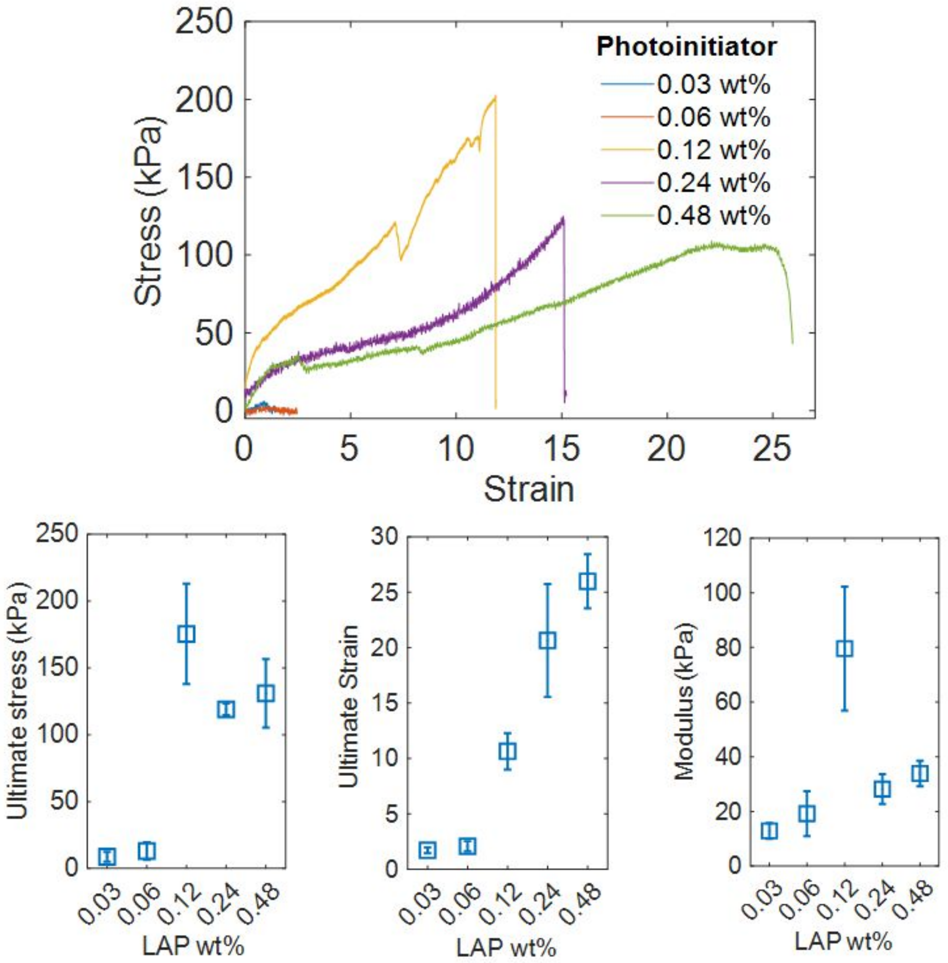

Figure S5. Stress-strain plot obtained from the TOPS printed hybrid gel structure with varying proportions of photoinitiator. Ultimate stress, ultimate strain, and elastic moduli obtained for the DN gel structures with varying concentrations of photoinitiator. (Error bars: Mean±SD)

Effect of composition of crosslinker on the tensile properties of TOPS printed DN gel dog-bone structures

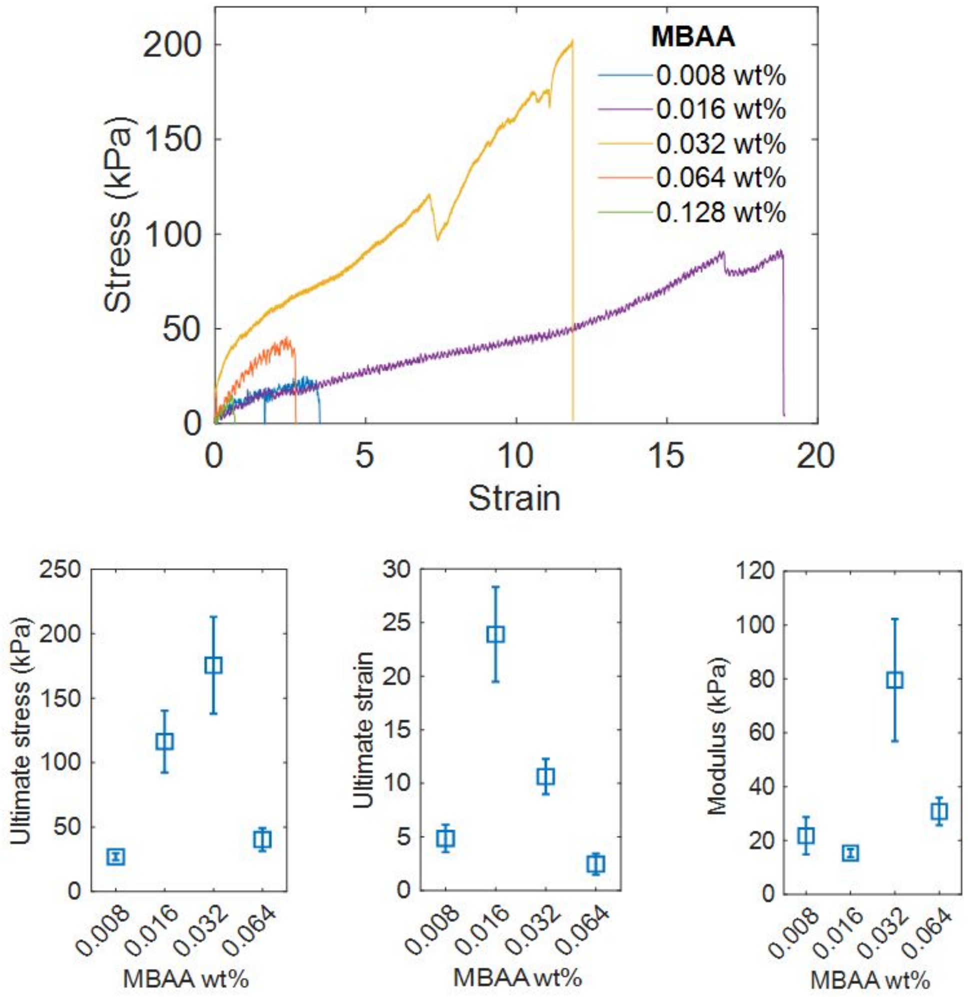

Figure S6. Stress-strain plots obtained from the TOPS printed hybrid gel structures printed with varying proportions of MBAA crosslinker (0.008 wt%, 0.016 wt%, 0.032 wt%, 0.064 wt%, and 0.128 wt%,). Ultimate stress, ultimate strain, and elastic moduli for the structures printed with varying amounts of MBAA crosslinker. (Error bars: Mean±SD)

Effect of composition of κ-carrageenan on the tensile properties of TOPS printed DN gel dog-bone structures

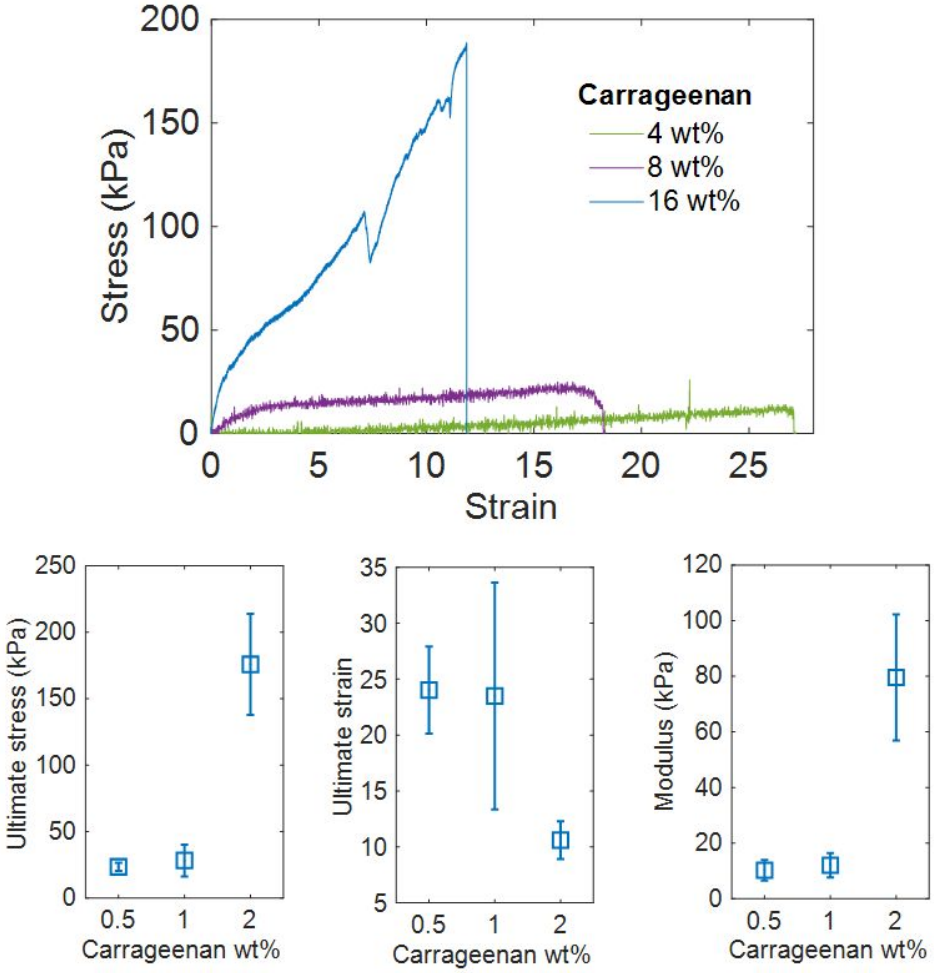

Figure S7. Stress-strain plots obtained from the hybrid structures printed with varying proportions of κ-carrageenan (0.5 wt%, 1 wt%, and 2 wt%). (Error bars: Mean±SD)

Swelling of DN gel structures

DN cylindrical stubs printed via TOPS were immersed in DI water. Results show that stub diameters and heights increased by 21% during the first hour, reached 45% in one day, and saturated after 78 hrs (**Figure S8**). In terms of mass, the printed structure (right after the printing) weighed 0.5 gm and the structure absorbed 7.9 gm of water, which is 17 times the original mass. Total water content before swelling was 81% and this increased to 98.8% after immersing the structure in water for 78 hours.

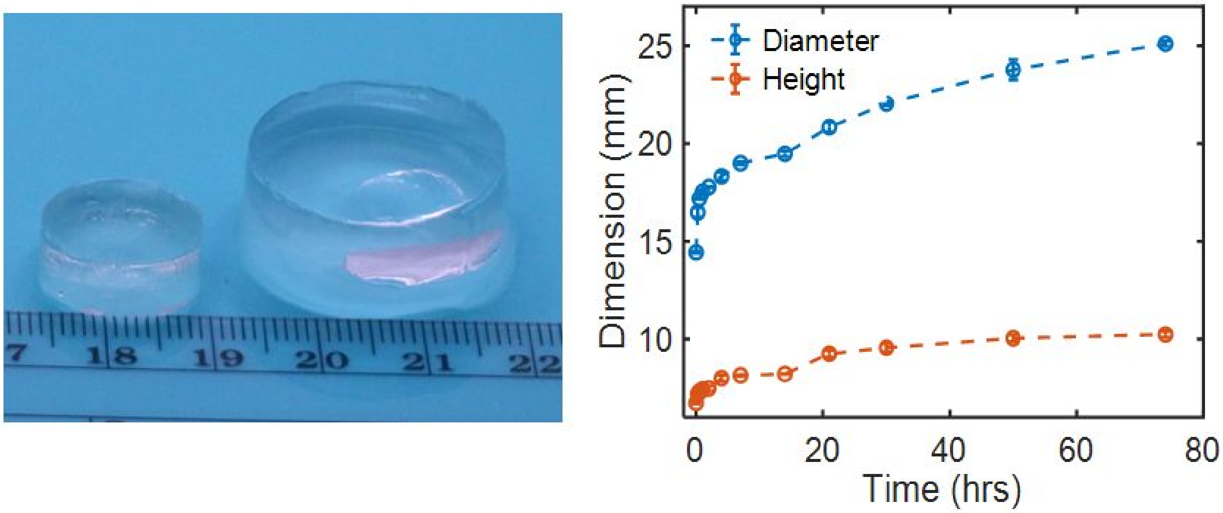

Figure S8. Image and plot of swelling of TOPS printed DN gels cylindrical stud and associated dimension (Diameter and Height) recorded for 78 hours.

Influence of swelling on tensile and compression properties

Tensile properties of DN dogbone samples swollen for 5 min, 10 min, 4hrs, and 4 days were studied. The 4-day swollen sample was too soft to reliably handle, and hence it was omitted from this study. Results show that the ultimate stress, the ultimate strain, and the modulus were highest for samples swelled for 5 minutes as compared to samples swelled for 10 minutes and 4 hours.

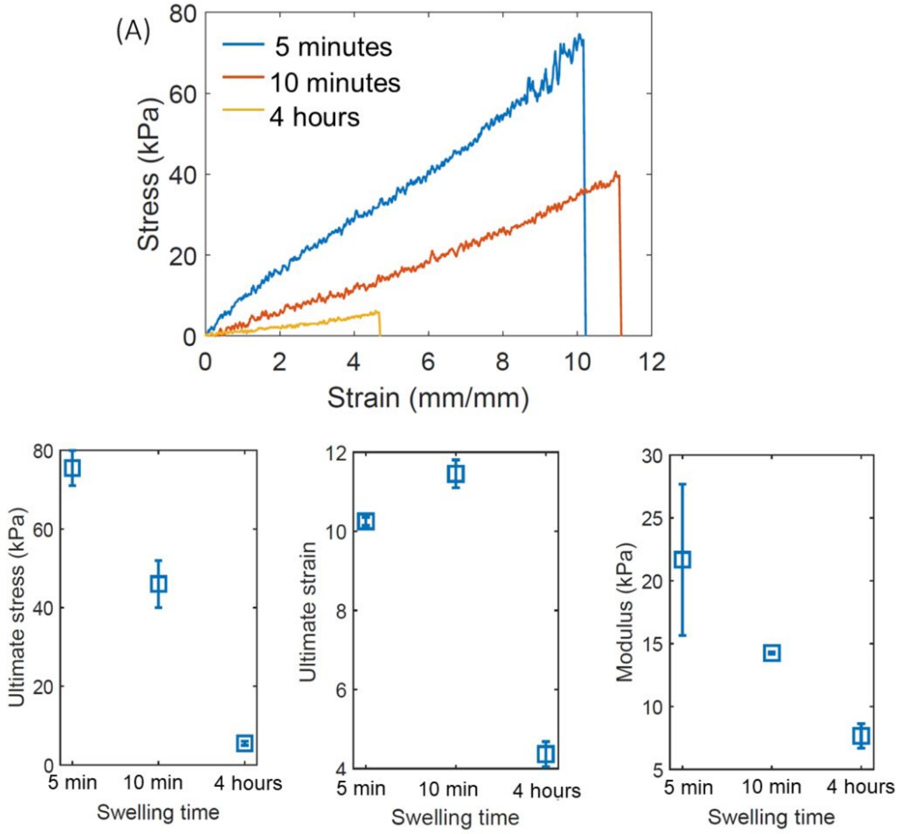

Figure S9. (A) Stress-strain curves recorded from the hybrid gel structures swelled for 5 minutes, 10 minutes, and 4 hours. Ultimate stress, ultimate strain, position, and elastic moduli for the hybrid structures swelled for different time points. (Error bars: Mean±SD)

Effect of exposure time on the tensile properties of swelled DN gel structure

Longer exposure time during TOPS printing resulted in lesser swelling and therefore exhibited better mechanical properties. For instance, structures exposed for 2 minutes swelled 1.28 times of original length in 4 hours, whereas the structure exposed for 1 minute swelled 1.85 times its length at the same time. Longer light exposure during TOPS, when swelled for 4 hours withstood the larger ultimate stress of 14±2 kPa and modulus 14.95±3.7 kPa. These parameters were 5.5±0.5 kPa and 7.68±0.9 kPa for the structure exposed for 1 minute. Further, the strain was almost double (8.05±1.45) for longer exposed structures (**Figure S10**).

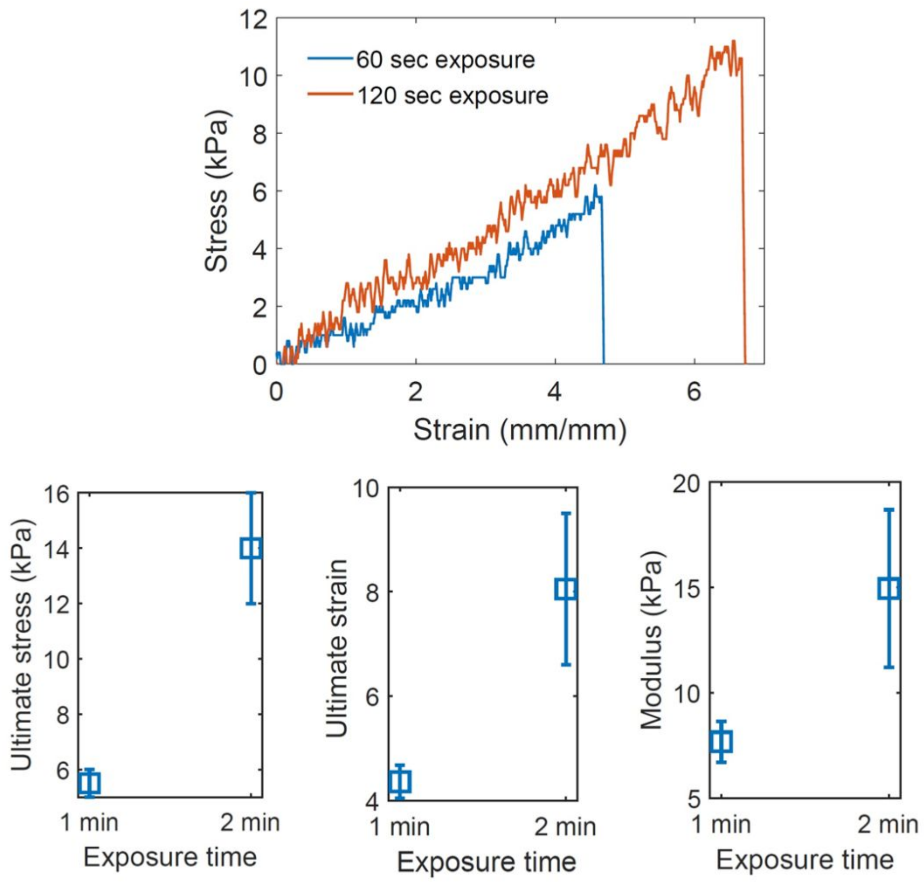

Figure S10. Stress-strain plots obtained from the swelled structures printed using different exposure times (60 seconds and 120 seconds). Structures were swelled for 4 hours in water. Ultimate stress, ultimate strain, position, and elastic moduli obtained from swelled structures printed using different exposure times.

Effects of hydration, dehydration, and rehydration

The ability of the printed structure to recover after dehydration followed by rehydration was tested. As-printed DN dogbone structure, dried for 2 days using a dehumidifier, was rehydrated in water for 30 mins until the size reaches 1.3 times the size of the as-printed structure. Tensile tests showed that these samples regained their ultimate stress (24.5±1.5 kPa), ultimate strain (7.675±0.205). Similar results were obtained when the as-printed samples were first hydrated completely, dehydrated completely, and rehydrated to 1.3 times the size of as-printed samples with ultimate stress (24±0 kPa), ultimate strain (9±0.6), and modulus (11.11±0.12 kPa) (**Figure S11**).

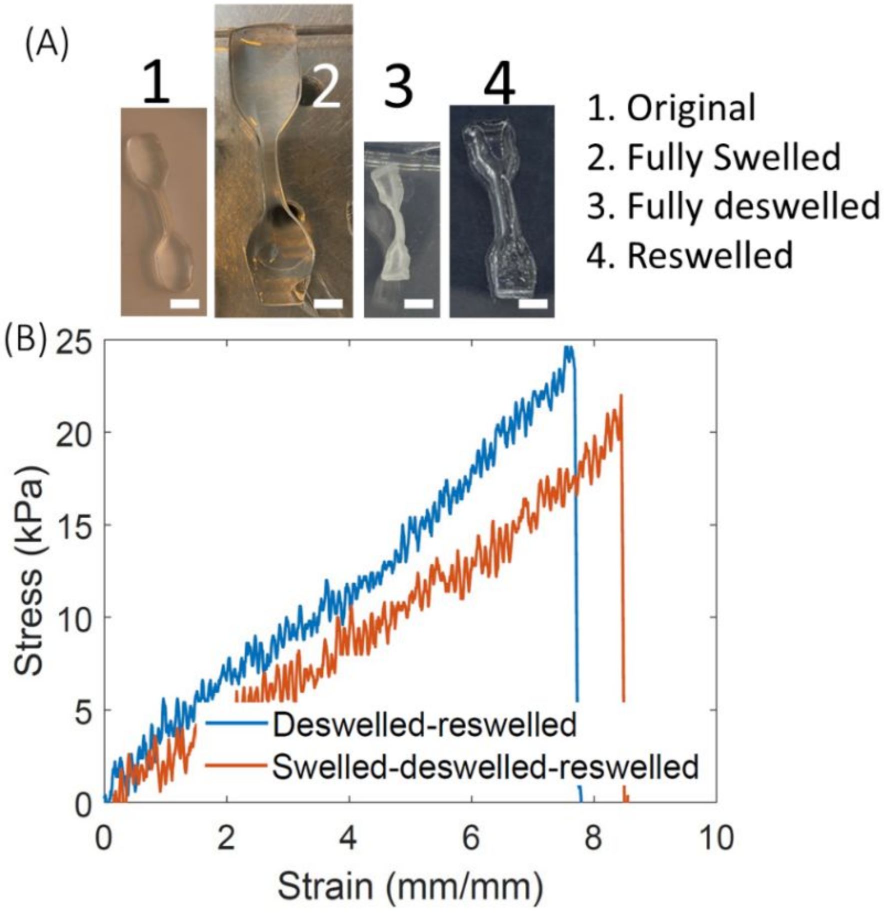

Figure S11. (A) Images of dog-bone structures after swelling and deswelling. (B) Stress-strain plots were obtained from the structures after different stages of swelling and deswelling. (Scale bar= 5mm)

Table S2. Assignment of Relevant FTIR fingerprints associated with functional groups obtained from Figure 4C(i).

Wavenumber (cm^{-1}), PAAm	Wavenumber (cm^{-1}), DN gel (Tensile Unloaded)	Assignment
1435	1437	ν, C─N
1454	1456	δ, C─H
1600	1598	δ, NH2 (amide II)
1668	1672	ν, C═O (amide I)

Self-healing of the 3D-printed structures

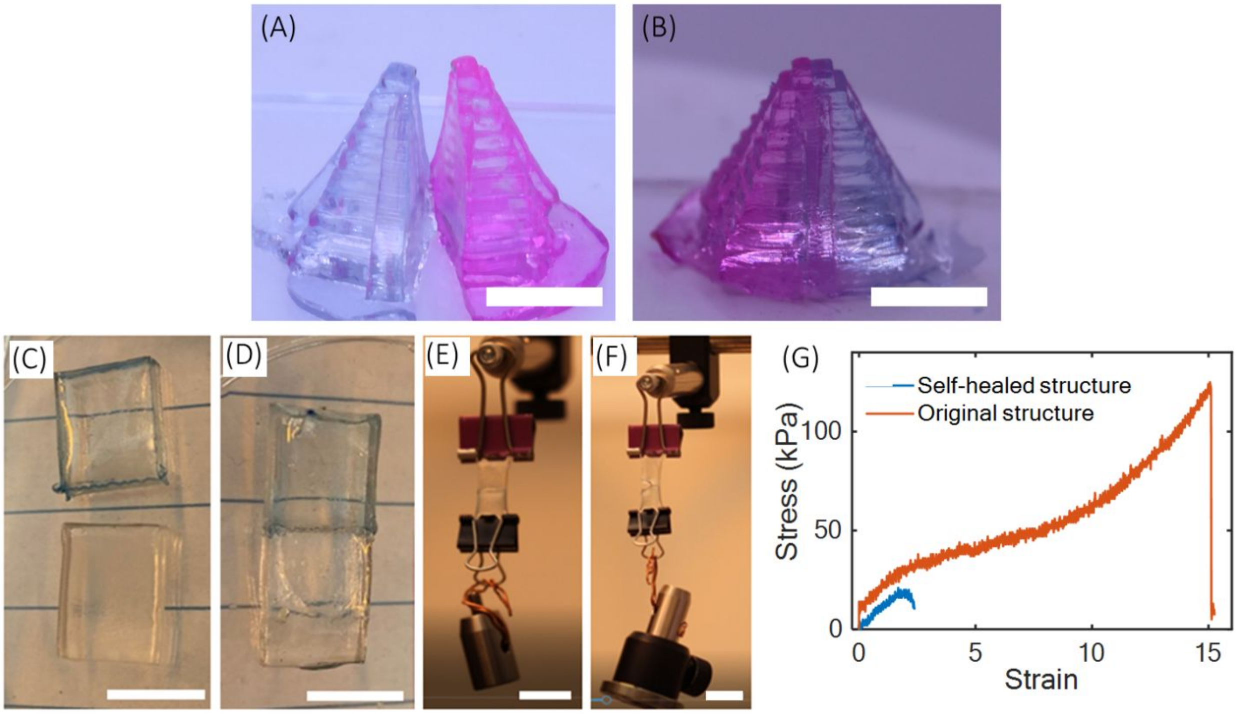

Figure S12. (A,B) Demonstration of self-healing properties of 3D printed Mayan pyramid structure (Scale bar- 5 mm). (C,D) Self-healing of rectangular slab printed using acrylamide/-carrageenan structure (scale bar- 10 mm). (E,F) Performance of self-healed structure subjected to a load of (70 gm) and (200 gm) (Scale bar-10mm) (G) stress-strain plot comparing the tensile performance of a self-healed structure with that of the original structure.

Thermogravimetric analysis (TGA) studies on DN gels structures

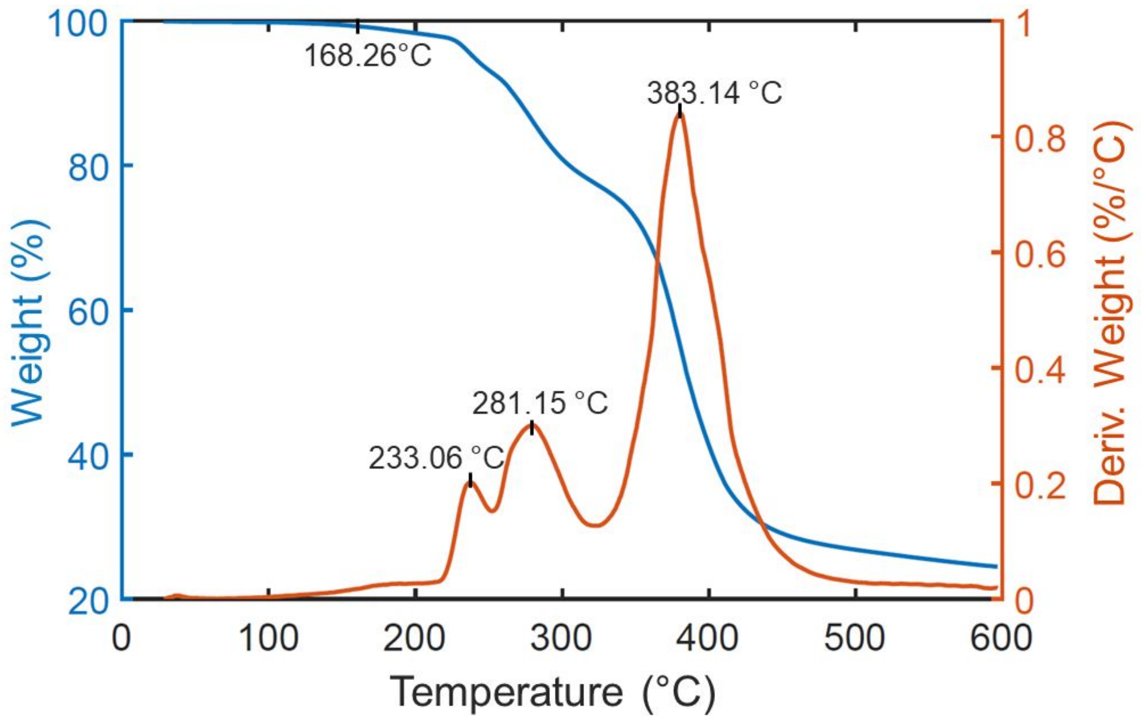

Figure S13. TGA plot obtained from fully dehydrated DN gel under a nitrogen atmosphere between 25˚C and 600˚C with a heating rate of 10˚C/min.

Effect of swelling on the compressive properties of DN gel structures

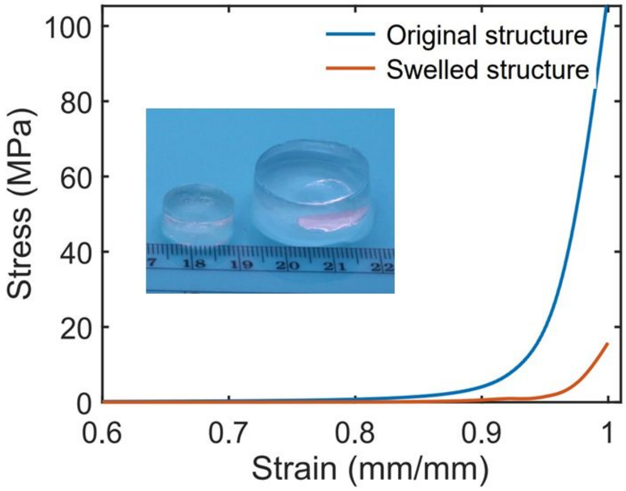

Figure S14. Stress-strain plot was obtained by compressing the original structure and the structure swelled for 4 days. Inset shows the original structure and swelled structure.

Lens stretcher

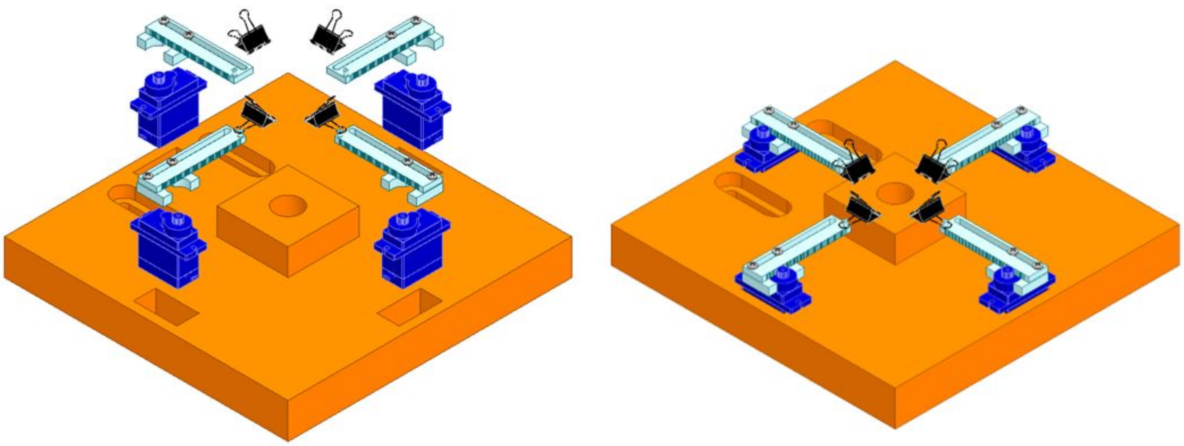

Figure S15. CAD design of before and after assembly of axicon lens stretching device.

References

[1] B. D. Fairbanks, M. P. Schwartz, C. N. Bowman, K. S. Anseth, *Biomaterials* **2009**, *30*, 6702.

Legends of supplementary movies

Movie V1. *Stretching of TOPS printed acrylamide/κ-carrageenan double network hydrogel dog-bone structure. This movie shows the stretching of structures with necking and without necking.*

Movie V2. *Stretching of TOPS printed acrylamide/κ-carrageenan double network hydrogel 3D structure.*

Movie V3. *Compression of TOPS printed Mayan Pyramid using acrylamide/κ-carrageenan double network hydrogel. This movie shows that the structure regains its structure after compression force is lifted.*

Movie V4. *Tunability of the axicon lens using a custom-designed lens stretcher. This movie shows a change in the size annular rings of the Bessel beam when the lens is stretched.*
